# Natural dolomitic limestone-catalyzed synthesis of benzimidazoles, dihydropyrimidinones, and highly substituted pyridines under ultrasound irradiation

**DOI:** 10.3762/bjoc.16.156

**Published:** 2020-08-03

**Authors:** Kumar Godugu, Venkata Divya Sri Yadala, Mohammad Khaja Mohinuddin Pinjari, Trivikram Reddy Gundala, Lakshmi Reddy Sanapareddy, Chinna Gangi Reddy Nallagondu

**Affiliations:** 1 Department of Chemistry, Green and Sustainable Synthetic Organic Chemistry Laboratory, Yogi Vemana University, Kadapa-516 005, Andhra Pradesh, India; 2 Department of Physics, S.V.D. College, Kadapa-516003, Andhra Pradesh, India

**Keywords:** benzimidazoles, dihydropyrimidinones, highly substituted pyridines, natural dolomitic limestone, ultrasound irradiation

## Abstract

Natural dolomitic limestone (NDL) is employed as a heterogeneous green catalyst for the synthesis of medicinally valuable benzimidazoles, dihydropyrimidinones, and highly functionalized pyridines via C–N, C–C, and C–S bond formations in a mixture of ethanol and H_2_O under ultrasound irradiation. The catalyst is characterized by XRD, FTIR, Raman spectroscopy, SEM, and EDAX analysis. The main advantages of this methodology include the wide substrate scope, cleaner reaction profile, short reaction times, and excellent isolated yields. The products do not require chromatographic purification, and the catalyst can be reused seven times. Therefore, the catalyst is a greener alternative for the synthesis of the above N-heterocycles compared to the existing reported catalysts.

## Introduction

**Figure 1:**
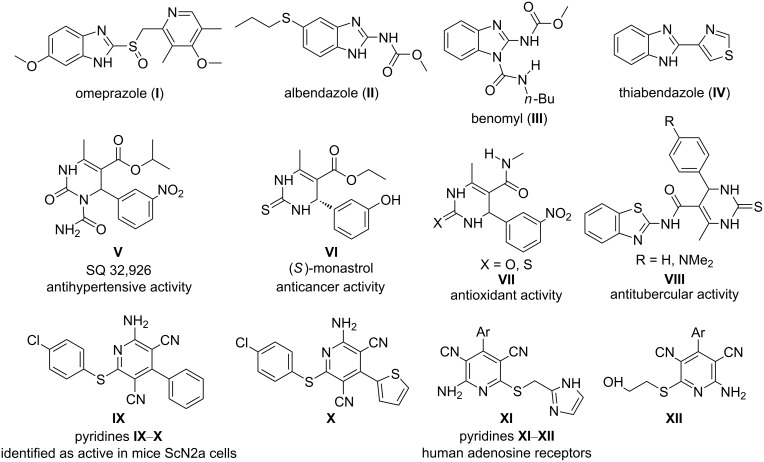
The benzimidazoles **I**–**IV**, dihydropyrimidinones/-thiones **V**–**VIII**, and 2-amino-4-aryl-3,5-dicarbonitrile-6-sulfanylpyridines **IX**–**XII** as medicinally privileged structures.

Nitrogen heterocycles are recognized as “privileged medicinal scaffolds” because these compounds are found in a wide variety of bioactive natural products and pharmaceuticals [1–3]. Among them, benzimidazoles, dihydropyrimidinones, and pyridines have emerged as promising and valuable structural units in many pharmaceutical lead compounds (Figure 1) [4–9]. Hence, there is a great need for the development of a green and sustainable synthetic route to the aforesaid nitrogen-containing heterocycles.

Benzimidazoles are an important class of N-heterocycles due to their potential applications in both biology and medicinal chemistry [10–13]. These compounds are used in the treatment of diseases, such as obesity, ischemia-reperfusion injury, hypertension, etc. [14–16]*.* In addition, these compounds are important intermediates in a variety of organic reactions and key elements in many functional materials [17–19]. Because of their potential utility, a huge number of synthetic protocols has been developed for the preparation of benzimidazole derivatives. The most common method for the preparation of benzimidazoles is the reaction between *o*-phenylenediamines and carboxylic acids [20–21]. Another general synthetic route reported is the condensation reaction of *o*-phenylenediamine with aldehydes in the presence of various catalysts, such as Zn–proline, trimethylsilyl chloride (TMSCl), Amberlite^®^ IR-120, indion 190, trifluoroethanol, YCl_3_, HClO_4_–SiO_2_, MMZ_Y_ zeolite, Er(OTf)_3_, etc. [22–30].

Developments in already established multicomponent reactions (MCRs) are interesting topics in organic synthesis. For instance, the Biginelli reaction is a renowned and tunable MCR to synthesize the pharmacologically active 3,4-dihydropyrimidin-2-(1*H*)-ones (DHPMs, Biginelli products) [31]. These compounds occupy an important position in the fields of natural products and synthetic organic chemistry owing to their potential pharmacological properties [32–37]. A wide variety of Brønsted acids and Lewis acids are employed as efficient catalysts for the Biginelli reaction [38–47]. In addition, some transition metal-based catalysts and a few nonacidic inorganic salts are also utilized as catalysts for the above reaction [48–58]. Only few basic catalysts, such as *t*-BuOK, Ph_3_P, and ʟ-proline are reported for the Biginelli reaction [59–61].

2-Amino-4-aryl-3,5-dicarbonitrile-6-sulfanylpyridines have gained considerable attention due to their wide-ranging biological activities [62–63]. The most common synthetic route for the preparation of 2-amino-4-aryl-3,5-dicarbonitrile-6-thiopyridines is the condensation reaction of aldehydes, malononitrile, and thiols in the presence of a variety of catalysts [64–72]. Though the reported methods are efficient to provide the desired 1,2-disubstituted benzimidazoles, dihydropyrimidinones/-thiones and 2-amino-4-aryl-3,5-dicarbonitrile-6-sulfanylpyridines, there are still some drawbacks, which include the use of expensive catalysts, the preparation of the catalyst, long reaction times, the limited substrate scope, and complicated work-up processes; further, the products require chromatographic purification.

The mineral NDL is an irregular combination of calcium and magnesium carbonate. It is water-insoluble, environmentally benevolent, inexpensive, nontoxic, and abundant in nature. Further, dolomite is used as a heterogeneous green catalyst in very few organic transformations, such as Knoevenagel, Michael–Henry, and transesterification reactions [73–74]. To the best of our knowledge, there are no reports on the NDL-catalyzed synthesis of aforesaid N-heterocycles under ultrasonic irradiation (USI).

**Scheme 1:**
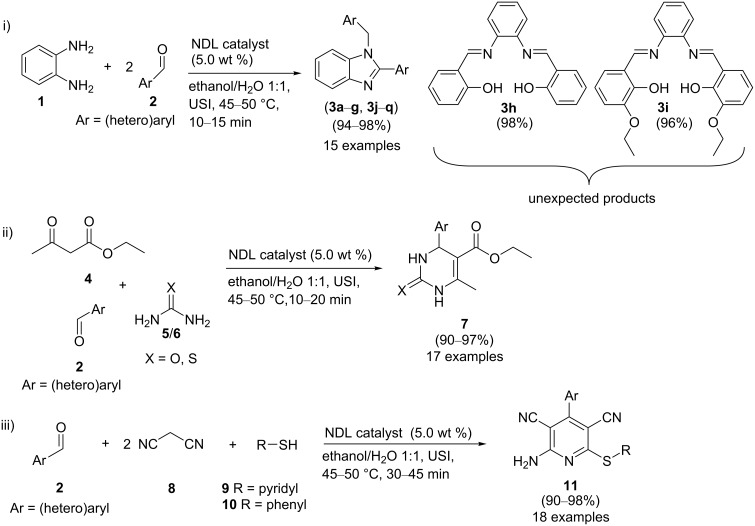
NDL-catalyzed synthesis of i) 1,2-disubstituted benzimidazoles **3**, ii) dihydropyrimidinones/-thiones **7**, and iii) 2-amino-4-(hetero)aryl-3,5-dicarbonitrile-6-sulfanylpyridines **11** under ultrasound irradiation.

In this paper, we wish to report the use of NDL as a heterogeneous green catalyst for the synthesis of the 1,2-disubstituted benzimidazoles **3**, the dihydropyrimidinones/-thiones **7**, and the 2-amino-4-(hetero)aryl-3,5-dicarbonitrile-6-sulfanylpyridines **11** via C–N, C–C, and C–S bond-forming reactions, respectively, in a mixture of EtOH and H_2_O 1:1 under ultrasonic irradiation (Scheme 1).

## Results and Discussion

### Geological background of the NDL catalyst

The NDL catalyst was collected from V. Kothapalli village (N 14°31’54”, E 78° 02’58”), Vemula Mandal of the Cuddapah district, Rayalaseema, Andhra Pradesh, India. The rock formation in the mineralized area of this village belongs to the Vempalli Formation (VF) of the Papaghni group of the lower Cuddapah Supergroup in the Cuddapah Basin (CB). The carbonate minerals, such as limestone and dolomite, are the most abundant ones and common sedimentary rocks present in this area.

### Catalyst characterization

The NDL catalyst was ground into a fine powder and then sieved in a 200-mesh sieve. The chemical composition of the catalyst was determined by standard quantitative analysis. The basic strength of the catalyst was analyzed by using Hammett indicators. The catalyst was characterized by XRD, IR, Raman, SEM, and EDAX analysis.

The chemical composition of the NDL was determined by adopting a standard quantitative analysis [75]. The obtained results are summarized in Table 1.

**Table 1:** Chemical composition of the NDL catalyst. LOI: loss of ignition.

component	LOI	CaO	MgO	SiO_2_	Al_2_O_3_	Fe_2_O_3_	SO_3_	Na_2_O	K_2_O

%	38.90	41.84	9.90	7.3	0.94	0.30	0.24	0.28	0.05

The basic strength of the NDL catalyst (H_) was measured using Hammett indicators, namely bromothymol blue (H_ = 7.2), phenolphthalein (H_ = 9.8), 2,4-dinitroaniline (H_ = 15.0), and nitroaniline (H_ = 18.4) as Hammett indicators. In each case, 5 mL of a methanolic solution of the Hammett indicator was added to 50 mg of the catalyst, shaken well, and then allowed to equilibrate for 2 h. No color variation of the indicators was observed. The study revealed that the basic strength of the NDL catalyst was weaker than the bromothymol blue indicator, i.e., H_ < 7.2. Hence, the NDL catalyst is a mild base, and it can activate both nucleophilic and electrophilic groups [73]. Further, the amount of basic sites on the catalyst was estimated by titration using a standard benzoic acid solution and bromothymol blue indicator. Initially, the catalyst (50 mg) was stirred with the methanolic solution of the indicator (5 mL) for 30–40 min, and then, the mixture was titrated with a 0.02 M benzoic acid solution. From the titer values of the benzoic acid solution, the amount of the basic sites was found to be 0.033 mmol/g.

**Figure 2:**
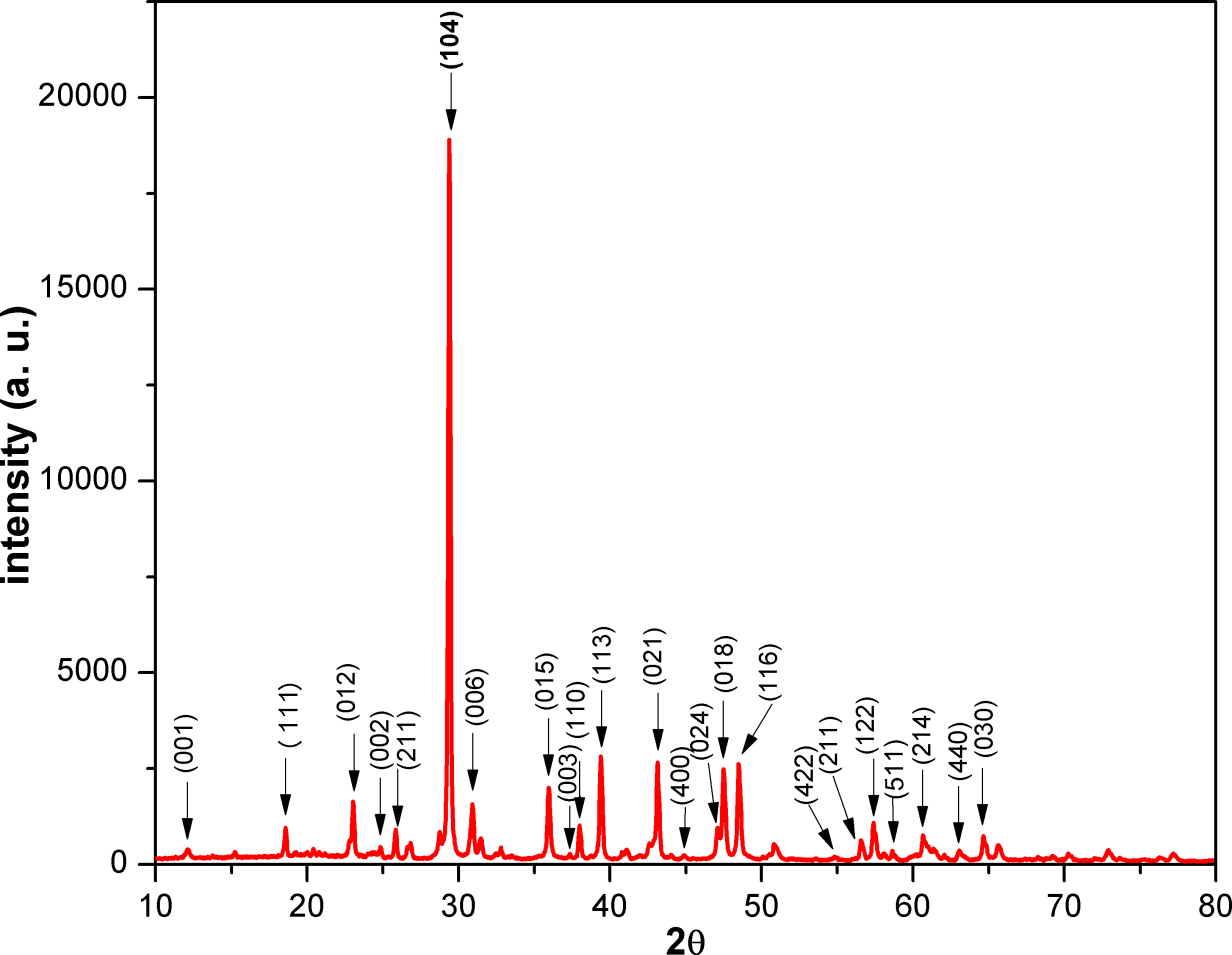
XRD pattern of the NDL catalyst.

The powder XRD pattern of the NDL catalyst is shown in Figure 2. The diffraction peaks at 2θ = 23.16, 29.51, 31.05, 36.02, 38.07, 39.40, 43.0, 47.2, 47.5, 48.5, 56.6, 57.6, 60.9, and 64.8° were attributed to the (012), (104), (006), (015), (110), (113), (021), (024), (018), (116), (211), (122), (214), and (030) plane, respectively, of the NDL catalyst (JCPDS card file 5–586: calcite and 11–78: dolomite) [76–77]. Small quantities of aluminium silicates (kaolinite) and iron oxides were also confirmed by the XRD pattern. The less intense diffraction peaks at 2θ = 12.3, 24.8, and 37.4 were assigned to the 001, 002, and 003 plane, respectively, of kaolinite (JCPDS card file 14-0164) [78]. The low-intense peaks at 2θ = 18.6, 26.1, 44.7, 54.6, 58.4, and 63.0 were ascribed to the 111, 211, 400, 422, 511, and 440 plane, respectively, of iron oxides (JCPDS card file 39-1346 and JCPDS card file 19-629) [79–80]. The above results were supported by FTIR and Raman characterization studies of the catalyst (vide infra).

**Figure 3:**
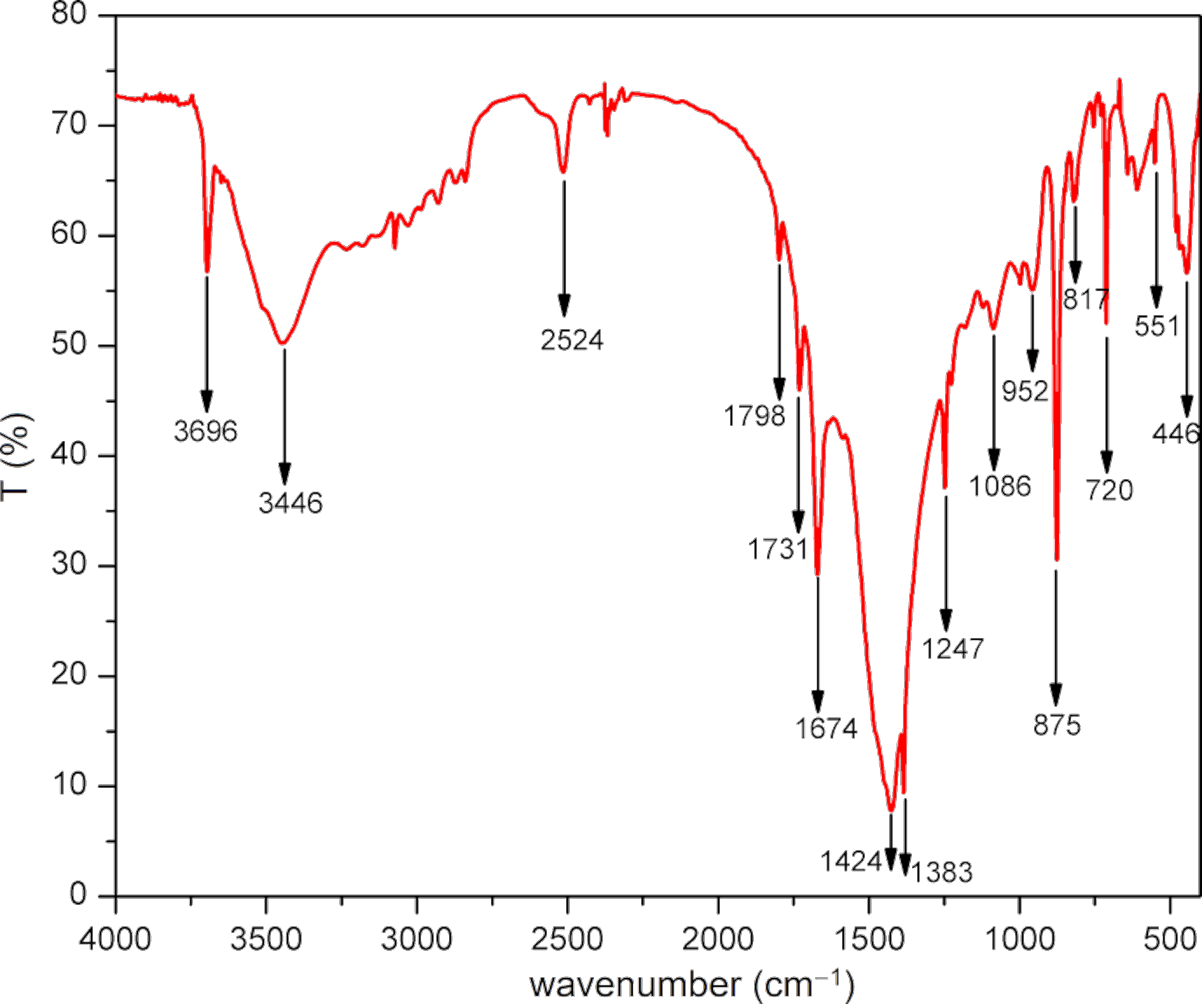
FTIR spectrum of the NDL catalyst.

The FTIR spectrum of the catalyst is shown in Figure 3. In the IR spectrum, two distinct vibrational modes of the carbonates, i.e., out-of-plane bending and in-plane bending, were observed at 875 cm^−1^ (ν_2_) and 720 cm^−1^ (ν_4_), respectively. The bands at 1086 cm^−1^ and 1424 cm^−1^ were ascribed to a symmetric stretching vibration (ν_1_) and an asymmetric stretching vibration (ν_3_) of the carbonate group, respectively. The combined bands of the carbonate group, i.e., ν_1_ + ν_4_ and ν_1_ + ν_3_ were observed at 1798 and 2524 cm^−1^, respectively [76–77,81]. The IR bands at 3446 cm^−1^ (broad) and 1674 cm^−1^ (sharp) indicated the presence of stretching and bending vibrations of water [82]. The impurities aluminium silicate and iron oxides in the NDL were confirmed by IR spectroscopy. The peaks located at 446, 551, 817, 952, 1247, and 1383 cm^−1^ were attributed to the Si–O bending, Fe–O stretching, Al–O–Si stretching, Si–OH bending, Si–O stretching, and Al–O bending, respectively [83–84]. Further, the sharp band at 3696 cm^−1^ indicated the presence of a well-ordered kaolinite structure [76].

**Figure 4:**
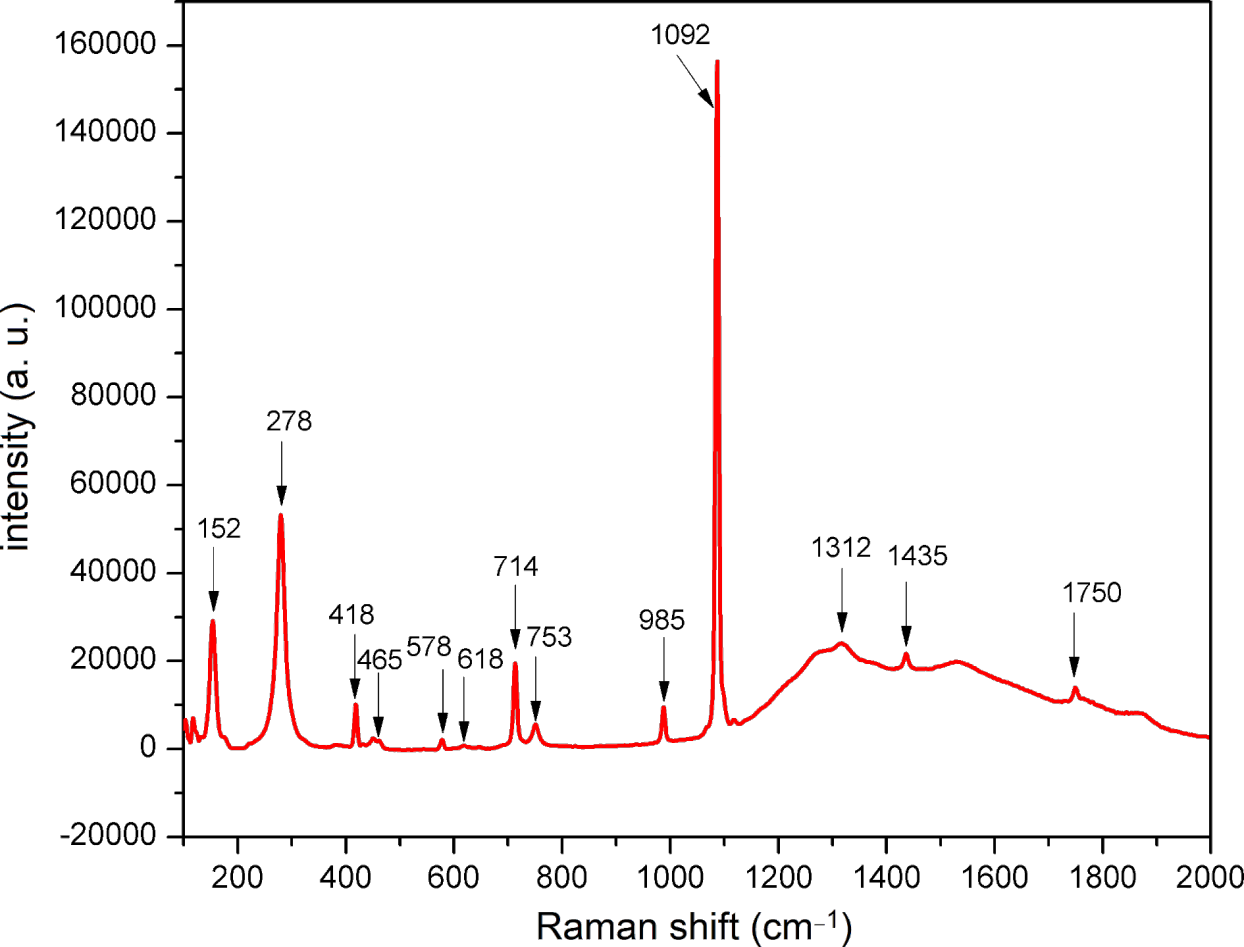
Raman spectrum of the NDL catalyst.

The Raman spectrum of the NDL catalyst is shown in Figure 4. The band at 1092 cm^−1^ was attributed to the symmetric stretching vibration (ν_1_) of the carbonate group. The peaks at 714 and 1435 cm^−1^ were assigned to a symmetric bending (ν_4_) and an asymmetric stretching vibration (ν_3_) of carbonate. The weak peak at 1750 cm^−1^ was due to the combined band ν_1_ + ν_4_. The bands at 152 and 278 cm^−1^ were ascribed to the external vibrations of the carbonate group [76–77]. The presence of aluminium silicates and iron oxides present in the sample were confirmed by Raman spectroscopy. The bands at 418, 578, 753, and 985 cm^−1^ were assigned to Al–O bending, Si–O rocking, Al–O stretching, and Si–OH stretching vibrations, respectively [85]. Further, a very weak peak at 618 cm^−1^ was attributed to iron oxide, and a very broad peak at 1312 cm^−1^ (magnon) indicated the presence of magnetically ordered ferromagnetic or antiferromagnetic iron oxides [86]. The observed Raman and infrared vibrational bands of the NDL were in good agreement with the reported values. The minor shift in the band positions might be due to the presence of trace metal contents and impurities.

**Figure 5:**
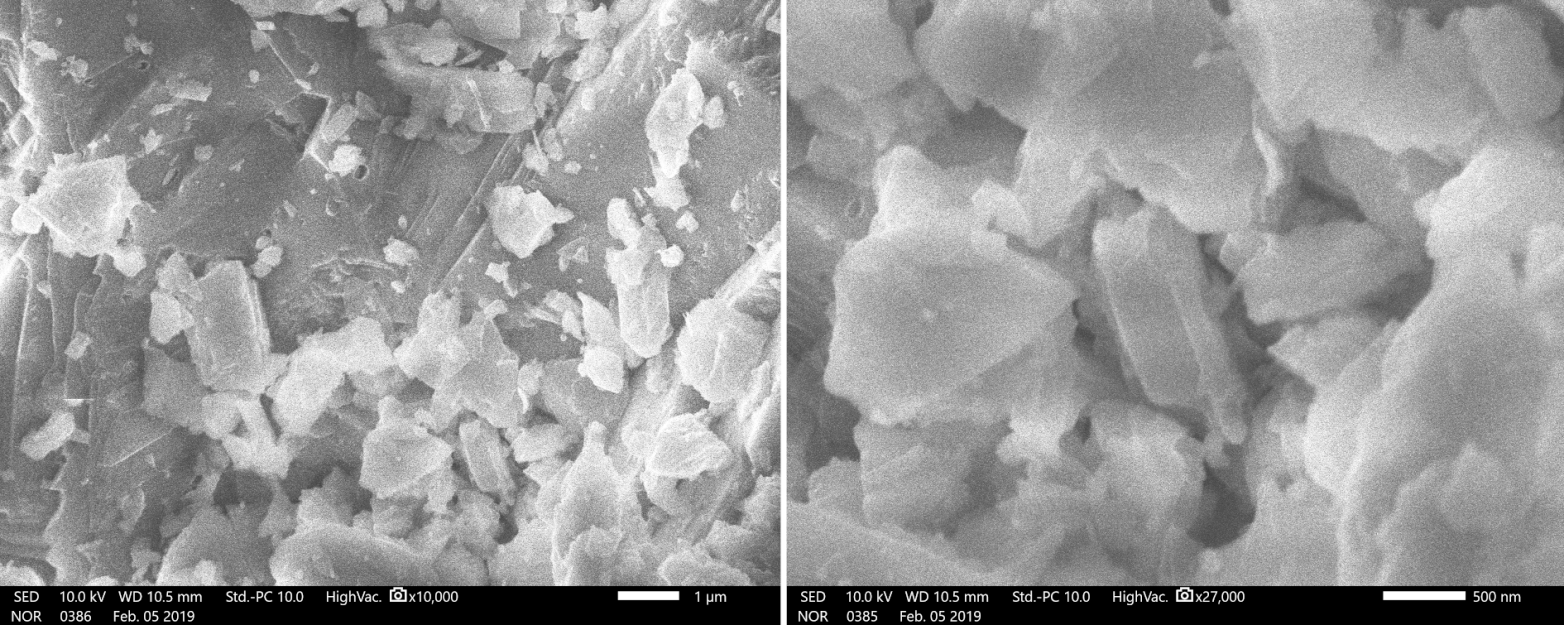
SEM images of the NDL catalyst.

**Figure 6:**
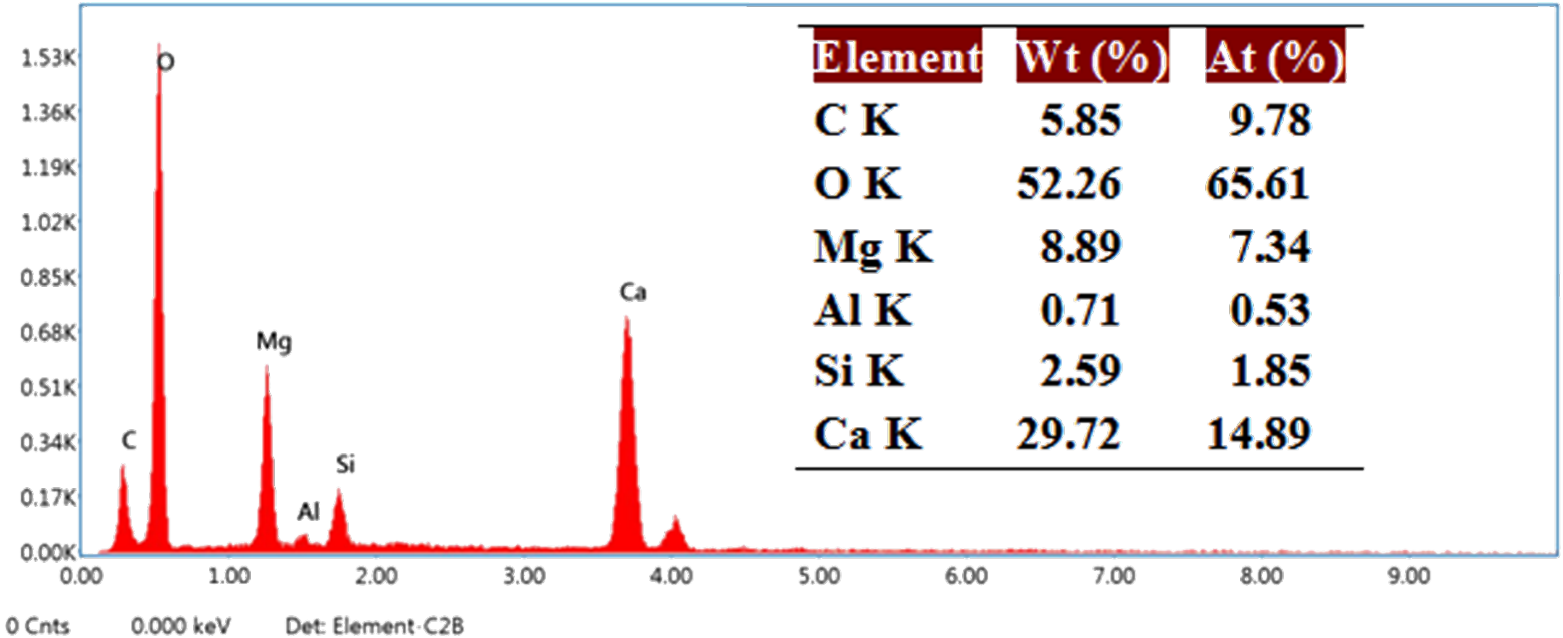
EDAX analysis of the NDL catalyst.

The morphology of the NDL catalyst was analyzed by scanning electron microscopy (Figure 5). The SEM images revealed that the morphology of the NDL catalyst consists of irregular shapes and sizes with a random dispersion. Further, the elemental composition of the NDL catalyst was determined by EDAX analysis (Figure 6).

The catalytic activity of the NDL for the synthesis of the 1,2-disubstituted benzimidazoles **3**, the dihydropyrimidinones/-thiones **7**, and the 2-amino-4-(hetero)aryl-3,5-dicarbonitrile-6-sulfanylpyridines **11** was investigated, along with other, commercially available catalysts.

### NDL-catalyzed synthesis of 1,2-disubtituted benzimidazoles **3**

To check the catalytic activity of the NDL, initially, *o*-phenylenediamine (**1**) and benzaldehyde (**2a**) were chosen as model substrates to optimize the reaction conditions for the synthesis of 1-benzyl-2-phenyl-1*H*-benzo[*d*]imidazole (**3a**). At first, a control experiment was conducted by using model substrates, **1** and **2a**, in H_2_O in the absence of catalyst under ultrasound irradiation for 60 min at 45–50 °C. It was found that the reaction did not proceed in the absence of a catalyst (Table 2, entry 1). To achieve the target compound **3a**, the same reaction was repeated by employing various catalysts (2.5 wt %), such as Fe_2_O_3_, Al_2_O_3_, KF–alumina, dolomitic limestone, triethylamine, pyridine, and DABCO in different solvents, such as water, acetone, iPrOH, EtOH, and EtOH/H_2_O 1:1 (Table 2, entries 2–8) under ultrasound irradiation at 45–50 °C. From this study, it was observed that the NDL (2.5 wt %) was the best option, which gave the target compound **3a** in a high yield (85%) in a mixture of EtOH and H_2_O 1:1 under ultrasound irradiation for 30 min at 45–50 °C (Table 2, entry 5). The other catalysts, Fe_2_O_3,_ Al_2_O_3,_ KF–alumina, triethylamine, pyridine, and DABCO, provided a moderate to low yield of the product **3a** (Table 2, entries 2–4 and 6–8). The aforesaid reaction was performed under conventional stirring of the model substrates **1** and **2a** in H_2_O in the absence of catalyst for 180 min at 45–50 °C. It was observed that the reaction did not proceed in the absence of a catalyst (Table 2, entry 1). Further, when the reaction temperature was raised from 45–50 °C to reflux, a very low yield (10%) of the product **3a** was obtained after 120 min. Next, the reaction was repeated in the presence of different catalysts and solvents at reflux under conventional reaction conditions as mentioned in Table 2. The study revealed that the NDL in a mixture of EtOH and H_2_O 1:1 afforded a moderate yield (70%) of the product **3a** (Table 2, entry 5), whereas the other catalysts, in various solvents, provided lower yields under similar reaction conditions (Table 2, entries 2–4 and 6–8). From the above observations, it was concluded that the ultrasound irradiation method is better than the conventional method in giving the maximum yield of **3a**.

**Table 2:** Optimization of the reaction conditions.^a^

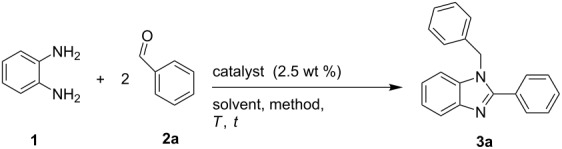							

entry	catalyst (2.5 wt %)	solvent	product	conventional method^b^		USI^c^	
				*t* (min)	yield^d^ (%)	*t* (min)	yield^d^ (%)

1^e^	no catalyst	H_2_O	**3a**	180	–	60	–
2	Fe_2_O_3_	H_2_O	**3a**	60	10	30	15
		acetone		60	–	30	–
		iPrOH		60	10	30	20
		EtOH		60	15	30	20
		EtOH/H_2_O 1:1		60	20	30	25
3	Al_2_O_3_	H_2_O	**3a**	60	20	30	20
		acetone		60	–	30	–
		iPrOH		60	15	30	20
		EtOH		60	25	30	25
		EtOH/H_2_O 1:1		60	30	30	40
4	KF–alumina	H_2_O	**3a**	60	30	30	30
		acetone		60	–	30	–
		iPrOH		60	25	30	30
		EtOH		60	40	30	35
		EtOH/H_2_O 1:1		60	50	30	40
5	NDL	H_2_O	**3a**	60	55	30	65
		acetone		60	–	30	–
		iPrOH		60	35	30	45
		EtOH		60	60	30	75
		EtOH/H_2_O 1:1		60	70	30	85
6	Et_3_N	H_2_O	**3a**	60	10	30	10
		acetone		60	–	30	–
		iPrOH		60	10	30	10
		EtOH		60	15	30	20
		EtOH/H_2_O 1:1		60	10	30	10
7	pyridine	H_2_O	**3a**	60	–	30	–
		acetone		60	–	30	–
		iPrOH		60	5	30	5
		EtOH		60	10	30	10
		EtOH/H_2_O 1:1		60	5	30	5
8	DABCO	H_2_O	**3a**	60	10	30	5
		acetone		60	–	30	–
		iPrOH		60	15	30	5
		EtOH		60	15	30	15
		EtOH/H_2_O 1:1		60	10	30	10

^a^Reaction conditions: *o*-phenylenediamine (**1**, 1.0 mmol), benzaldehyde (**2a**, 2.0 mmol), catalyst (2.5 wt %), solvent (3.0 mL). ^b^Performed by stirring at reflux (entries 2–8). ^c^USI method performed at 45–50 °C. ^d^Isolated yield. ^e^Conventional method performed by stirring at 45–50 °C.

Next, the amount of catalyst was varied (using 2.5, 5.0, 7.5, 10.0, and 12.5 wt %, respectively,) to improve the yield of **3a** (Table 3). The study revealed that 5.0 wt % of the NDL was the best option to get the highest yield of the product **3a** (98%) in a short reaction time (10 min, Table 3, entry 3). It was also noticed that the same yield was obtained with an increasing amount of the catalyst, i.e., 7.5, 10.0, and 12.5 wt % (Table 3, entries 4–6).

**Table 3:** Effect of the catalyst loading.^a^

entry	NDL (wt %)	solvent	*t* (min)	product	yield^b^ (%)

1	2.5	EtOH/H_2_O 1:1	30	**3a**	85
2	2.5	EtOH/H_2_O 1:1	10	**3a**	75
3	5.0	EtOH/H_2_O 1:1	10	**3a**	98
4	7.5	EtOH/H_2_O 1:1	10	**3a**	98
5	10.0	EtOH/H_2_O 1:1	10	**3a**	98
6	12.5	EtOH/H_2_O 1:1	10	**3a**	98

^a^Reaction conditions: *o*-phenylenediamine (**1**, 1.0 mmol), benzaldehyde (**2a**, 2.0 mmol), NDL (2.5 to 12.5 wt %), EtOH/H_2_O 1:1 (3.0 mL), ultrasound irradiation at 45–50 °C. ^b^Isolated yield.

In order to demonstrate the effect of the temperature on the course of the model reaction, the control experiment was performed at different temperature ranges (30–35, 35–40, 40–45, and 45–50 °C) by using the model substrates **1** and **2a** in the presence of 5.0 wt % of the NDL in a mixture of ethanol and water 1:1 for 10 min under both conventional stirring and ultrasound irradiation. The obtained results are presented in Table 4. It was observed that the reaction proceeded with an improved yield of **3a** (70–98%) by increasing the temperature range from 30–35 to 45–50 °C with an ultrasound irradiation method (Table 4, entries 1–4). Under conventional stirring, the yield of the product **3a** increased from low to moderate when the reaction temperature was raised from 30–35 °C to reflux (Table 4, entries 1–5). From the results, it was concluded that a temperature of 45–50 °C is the optimum temperature to obtain the maximum yield of the desired product **3a** within a short reaction time (10 min) under ultrasound irradiation (Table 4, entry 4).

**Table 4:** Effect of the temperature.^a^

entry	*T* (°C)	product	*t* (min)	conventional method^b^	USI^c^
				yield^d^ (%)	yield^d^ (%)

1	30–35	**3a**	10	10	70
2	35–40	**3a**	10	14	79
3	40–45	**3a**	10	20	87
4	45–50	**3a**	10	26	98
5^e^	reflux	**3a**	10/60	35/70	–

^a^Reaction conditions: *o*-phenylenediamine (**1**, 1.0 mmol), benzaldehyde (**2a**, 2.0 mmol), NDL (5.0 wt %), EtOH/H_2_O 1:1 (3.0 mL). ^b^Conventional stirring and heating with a silicone oil bath. ^c^Ultrasound irradiation in a water bath. ^d^Isolated yield. ^e^Conventional stirring at reflux.

To demonstrate the generality and substrate scope of the present method, a variety of (hetero)aromatic aldehydes was investigated. The obtained results are presented in Table 5. *o*-Phenylenediamine (**1**) reacted well with benzaldehyde (**2a**) to obtain the corresponding product **3a** with 98% yield (Table 5, entry 1). The reactions of *o*-phenylenediamine (**1**) with substituted benzaldehydes having activating groups (4-Me: **2b**, 4-*t*-Bu: **2c**, 2,4-dimethyl: **2d**, 4-OMe: **2e**, 3,4-dimethoxy: **2f**, 3,4,5-trimethoxy: **2g**, 4-OH-3-OMe: **2j**, and 4-OH-3-OC_2_H_5_: **2k**, Table 5, entries 2–7, 10 and 11), a deactivating group (4-NO_2_: **2l**, Table 5, entry 12), or a halo group (4-F: **2m**, 4-Cl: **2n**, and 4-Br: **2o**, Table 5, entries 13–15) in different positions provided good to excellent isolated yields of the corresponding products **3b**–**g** and **3j**–**o** that ranged from 94 to 98% in a stipulated period of time, as specified in Table 5. Further, heteroaromatic aldehydes, such as furan-2-aldehyde (**2p**) and thiophene-2-aldehyde (**2q**) produced the corresponding products **3p** and **3q** in good isolated yields within a short period of time (15 min and 13 min, respectively, Table 5, entries 16 and 17).

**Table 5:** NDL-catalyzed synthesis of 2-aryl-1-arylmethyl-1*H*-benzo[*d*]imidazoles 3.^a^

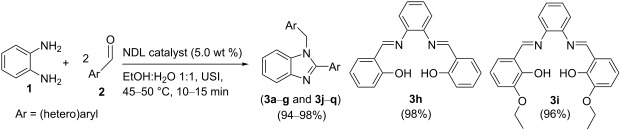						

entry	Ar	product	*t* (min)	yield^c^ (%)	mp (°C)	

					found	reported

1	phenyl: **2a**	**3a**	10	98	128–131	133–134 [23]
2	4-methylphenyl: **2b**	**3b**	10	98	127–128	128–129 [23]
3	4-*tert*-butylphenyl: **2c**	**3c**	15	94	124–125	122–126 [25]
4	2,4-dimethylphenyl: **2d**	**3d**	12	96	120–122	119–123 [25]
5	4-methoxyphenyl: **2e**	**3e**	11	98	157–159	158–160 [23]
6	3,4-dimethoxyphenyl: **2f**	**3f**	12	95	167–169	171–173 [24]
7	3,4,5-trimethoxyphenyl: **2g**	**3g**	15	94	261–262	262–263 [22]
8^b^	2-hydroxyphenyl: **2h**	**3h**	10	98	167–168	160–162 [23]
9^b^	2-hydroxy-3-ethoxyphenyl: **2i**	**3i**	12	96	285–287	–
10	4-hydroxy-3-methoxyphenyl: **2j**	**3j**	12	96	181–183	184–186 [24]
11	4-hydroxy-3-ethoxyphenyl: **2k**	**3k**	10	97	205–207	200–201 [26]
12	4-nitrophenyl: **2l**	**3l**	10	98	190–192	189–191 [23]
13	4-fluorophenyl: **2m**	**3m**	10	98	108–109	110–112 [23]
14	4-chlorophenyl: **2n**	**3n**	10	98	138–140	137–139 [23]
15	4-bromophenyl: **2o**	**3o**	12	96	158–160	160–162 [23]
16	2-furanyl: **2p**	**3p**	15	95	90–92	88–89 [23]
17	2-thienyl: **2q**	**3q**	13	96	149–150	150–152 [23]

^a^Reaction conditions: *o*-phenylenediamine (**1**, 1.0 mmol), aldehyde (**2**, 2.0 mmol), NDL (5.0 wt %), EtOH/H_2_O 1:1 (3.0 mL), USI, 45–50 °C. ^b^The reaction stopped at the bisimine I, i.e., **3h**/**i** stage. ^c^Isolated yield.

**Scheme 2:**
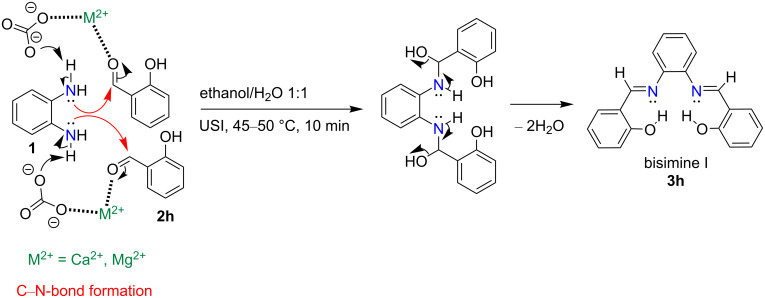
Unexpected formation of the bisimine I, **3h**, from *o*-phenylenediamine (**1**) and salicylaldehyde (**2h**).

**Figure 7:**
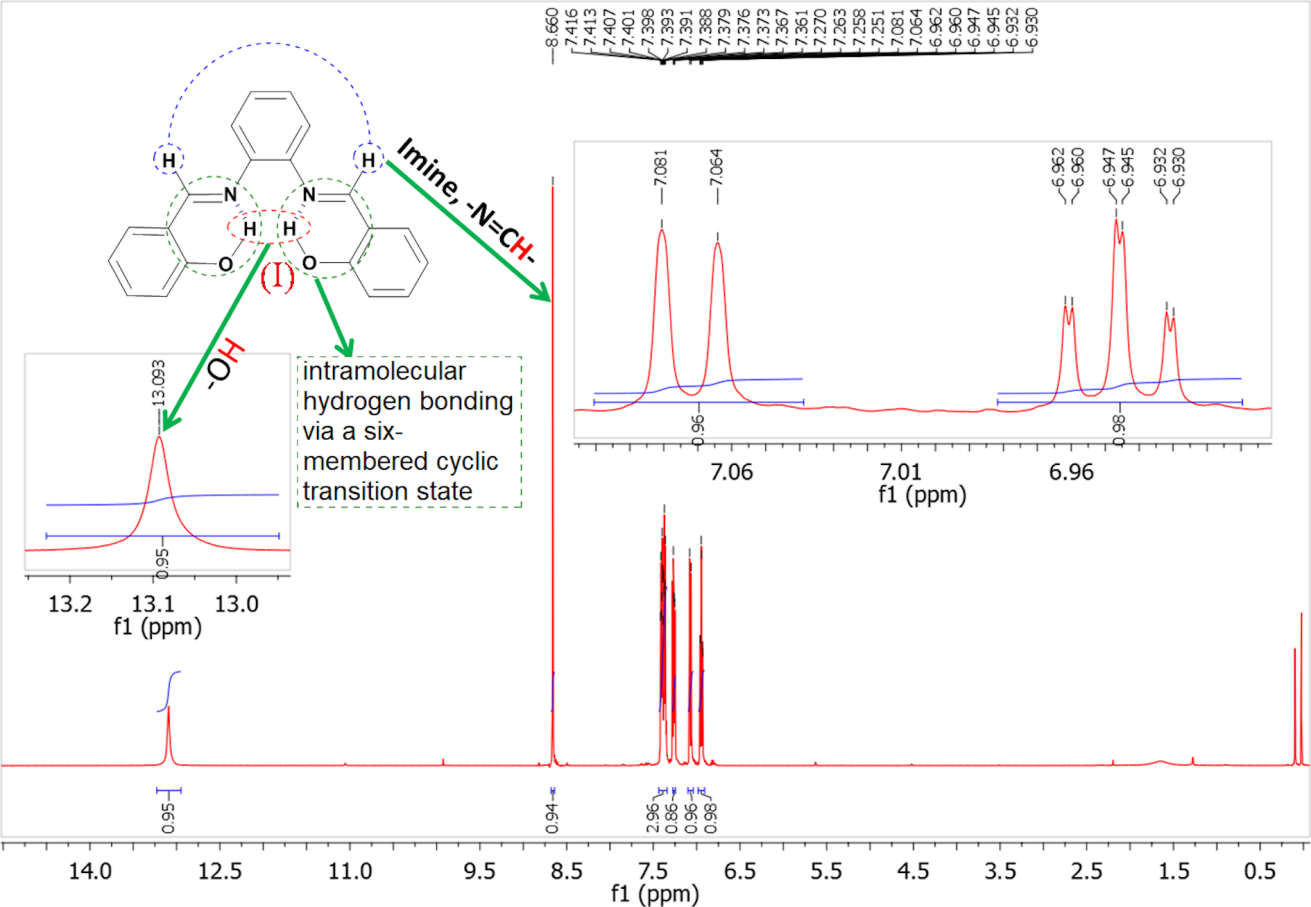
^1^H NMR spectrum of 2,2'-((1*E*,1'*E*)-(1,2-phenylenebis(azanylylidene))bis (methanylylidene))diphenol (bisimine I, **3h**).

However, salicylaldehyde (**2h**) afforded the unexpected product 2,2'-((1*E*,1'*E*)-(1,2-phenylenebis(azanylylidene))bis(methanylylidene))diphenol (**3h**, bisimine I) within 10 min (Table 5, entry 8). The reaction was expected to proceed through the activation of the carbonyl group of **2h** (of which 2.0 mmol were used) by the cations (Ca^2+^ and Mg^2+^, respectively) of the NDL. This was followed by a nucleophilic attack of the NH_2_ groups of *o*-phenylenediamine (**1**, of which 1.0 mmol was used), which are activated by the carbonate part of the NDL, followed by dehydration to obtain **3h** (Scheme 2). Due to the mild basic nature of the NDL catalyst, it acts as a dual activator of the electrophilic carbonyl and the nucleophilic NH_2_ groups. The formation of the bisimine I was confirmed by ^1^H NMR spectral studies (Figure 7). In the ^1^H NMR spectrum (DMSO-*d*_6_), the two hydroxy protons of the bisimine I appeared as a broad, strongly downfield-shifted singlet at δ 13.19. The sharp singlet at δ 8.66 indicated the two imine protons (–N=C**H**) of the bisimine I. From this result, it was confirmed that the reaction stopped at the bisimine I stage. This was due to the intramolecular hydrogen bonding between the hydrogen atom of the *ortho*-hydroxy group and the nitrogen atom of the imine group in a six-membered ring transition state [87]. Similarly, the reaction between 3-ethoxysalicylaldehyde (**2i**) and *o*-phenylenediamine (**1**) also ended with the intermediate 6,6'-((1*E*,1'*E*)-(1,2-phenylenebis(azanylylidene))bis(methanylylidene))bis(2-ethoxyphenol) (**3i**) stage (Table 4, entry 9 and Supporting Information File 1, Figure S13). Most of the synthesized compounds are known and were identified easily by comparison of the melting point and spectroscopic data with those reported.

### NDL-catalyzed synthesis of dihydropyrimidinones/-thiones **7**

The results encouraged us to further investigate the catalytic activity of the NDL in the Biginelli reaction. To check the feasibility, a control experiment was performed by using the model substrates benzaldehyde (**2a**, 1.0 mmol), ethyl acetoacetate (**4**, 1.0 mmol), and urea (**5**, 1.0 mmol) in H_2_O (3.0 mL) in the absence of a catalyst under ultrasound irradiation at 45–50 °C for 60 min. It was observed that the reaction proceeded with a very low yield (20%) of product **7a**. The same reaction was repeated in the presence of the NDL catalyst (5.0 wt %) in EtOH/H_2_O 1:1 under ultrasound irradiation at 45–50 °C for 15 min, which resulted in 97 % yield of **7a**.

To exploit the substrate scope and generality of the method, various (hetero)aromatic aldehydes **2** were examined. The obtained results are summarized in Table 6. Benzaldehyde (**2a**) underwent the reaction with ethyl acetoacetate (**4**) and urea (**5**) to obtain the corresponding dihydropyrimidinone **7a** in 97% yield (Table 6, entry 1). Benzaldehyde derivatives bearing electron-donating groups, such as 4-Me (**2b**), 4-OMe (**2e**), 3,4-dimethoxy (**2f**), 3-OH (**2r**), and 2-OH (**2h**), respectively, at different positions on the ring reacted well with ethyl acetoacetate (**4**) and urea (**5**) to produce the products, **7b**–**f** in good isolated yields that ranged from 92–96% (Table 6, entries 2–6). A benzaldehyde derivative with an electron-accepting nitro group (**2l**) at the *para* position on the ring showed a good reactivity with ethyl acetoacetate (**4**) and urea (**5**) to afford the product **7g** in an excellent isolated yield (94%, Table 6, entry 7). Halogen atoms at different positions on the ring of benzaldehyde derivatives (4-F: **2m**, 4-Cl; **2n**, and 3-Br: **2s**) underwent the reaction with ethyl acetoacetate (**4**) and urea (**5**) to form the corresponding products (**7h**–**j**) in good isolated yields that ranged from 93–96% (Table 6, entries 8–10). Heteroaromatic aldehydes, such as furan-2-aldehyde (**2p**) and thiophene-2-aldehyde (**2q**) showed a good reactivity, with good yields of **7k** (90%) and **7l** (92%), respectively (Table 6, entries 11 and 12). From this study, it was concluded that the optimized reaction conditions are suitable for monosubstituted (both electron-rich and electron-deficient) and disubstituted benzaldehyde derivatives as well as heteroaromatic aldehydes. To expand the scope of this method, thiourea (**6**) was also investigated (Table 6, entries 13–17). Benzaldehyde (**2a**) reacted with ethyl acetoacetate (**4**) and thiourea (**6**) to give the product **7m** in an excellent isolated yield (96%, Table 6, entry 13). Benzaldehyde derivatives bearing electron-donating groups, such as 4-Me (**2b**) and 4-OMe (**2c**) exhibited a good reactivity with ethyl acetoacetate (**4**) and thiourea (**6**) to produce the products **7n** (95%) and **7o** (95%) in excellent yields, respectively (Table 6, entries 14 and 15). Benzaldehyde with electron-withdrawing groups, such as 4-NO_2_ (**2f**) and 4-Cl (**2i**) at the *para* position reacted well with ethyl acetoacetate (**4**) and thiourea (**6**) to afford the corresponding products **7p** and **7q** in good isolated yields (94 and 95%) (Table 6, entries 16 and 17). Most of the synthesized compounds are known and were identified easily by comparison of the melting point and spectroscopic data with those reported.

**Table 6:** NDL-catalyzed synthesis of dihydropyrimidinone/-thione derivatives **7**.^a^

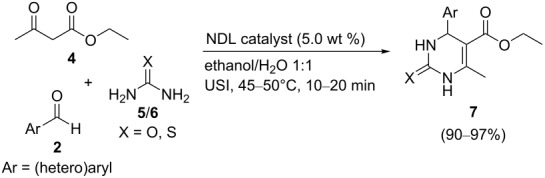							

entry	Ar	X	product	*t* (min)	yield^b^ (%)	mp (°C)	

						found	reported

1	phenyl: **2a**	O	**7a**	15	97	207–209	209–210 [50]
2	4-methylphenyl: **2b**	O	**7b**	15	96	213–214	215–216 [38]
3	4-methoxyphenyl: **2e**	O	**7c**	17	96	200–201	199–202 [48]
4	3,4-dimethoxyphenyl: **2f**	O	**7d**	18	94	213–215	212–214 [52]
5	3-hydroxyphenyl: **2r**	O	**7e**	17	95	162–164	163–165 [38]
6	2-hydroxyphenyl: **2h**	O	**7f**	15	92	198–200	199–201 [49]
7	4-nitrophenyl: **2l**	O	**7g**	12	94	210–211	209–212 [48]
8	4-fluorophenyl: **2m**	O	**7h**	13	95	176–179	175–177 [37]
9	4-chlorophenyl: **2n**	O	**7i**	12	96	208–210	209–211 [48]
10	3-bromophenyl: **2s**	O	**7j**	18	93	184–185	185–186 [47]
11	2-furanyl: **2p**	O	**7k**	20	90	204–206	203–205 [48]
12	2-thienyl: **2q**	O	**7l**	20	92	216–218	215–217 [38]
13	phenyl: **2a**	S	**7m**	15	96	211–212	208–210 [38]
14	4-methylphenyl: **2b**	S	**7n**	15	95	189–190	192–194 [38]
15	4-methoxyphenyl: **2e**	S	**7o**	17	95	148–150	150–152 [38]
16	4-nitrophenyl: **2l**	S	**7p**	10	94	113–114	109–111 [38]
17	4-chlorophenyl: **2n**	S	**7q**	11	95	190–191	192–194 [38]

^a^Reaction conditions: aldehyde (**2**, 1.0 mmol), ethyl acetoacetate (**4**, 1.0 mmol), urea/thiourea (**5/6**, 1.0 mmol), NDL (5.0 wt %), ethanol/H_2_O 1:1 (3.0 mL), USI at 45–50 °C. ^b^Isolated yield.

### NDL-catalyzed synthesis of 2-amino-4-(hetero)aryl-3,5-dicarbonitrile-6-sulfanylpyridines **11**

We further examined the catalytic efficacy of the NDL catalyst in the synthesis of the medicinally privileged highly functionalized pyridines **11**. For this purpose, a control experiment in the absence of a catalyst was conducted by using the model substrates benzaldehyde (**2a**, 1.0 mmol), malononitrile (**8**, 2.0 mmol), and 2-mercaptopyridine (**9**, 1.0 mmol) in H_2_O (3.0 mL) under ultrasound irradiation at 45–50 °C for 60 min. It was observed that the reaction did not afford any product in the absence of a catalyst. The above reaction was carried out in the presence of the NDL (5.0 wt %) in EtOH/H_2_O 1:1 (3.0 mL) under ultrasound irradiation for 10 min, which resulted in 70% yield of **11a**. To improve the yield of **11a**, the same reaction was repeated at different time intervals; 15, 20, 25, 30, 35, and 40 min, respectively, at 45–50 °C, and the yields of **11a** obtained were 75, 83, 89, 96%, 96, and 96%, respectively. From this study, it was found that the maximum yield of **11a** (96%) was obtained in 30 min, and the yields remained the same when the reaction time was increased from 30 to 40 min.

The optimized procedure was successfully applied for the synthesis of a series of highly substituted pyridines (**11b**–**r**, Table 7) by utilizing a range of (hetero)aromatic aldehydes **2**, malononitrile (**8)**, and the thiols **9** and **10**, respectively, as starting materials. Benzaldehyde (**2a**) underwent the reaction with malononitrile (**8**) and 2-mercaptopyridine (**9**) to form product **11a** in 96% yield (Table 7, entry 1). Benzaldehyde derivatives containing a range of functional groups, such as electron-donating groups (4-OMe: **2e**, 3,4,5-trimethoxy: **2g**, and 3-OH: **2r**), an electron-withdrawing group (4-NO_2_: **2l**), and halogen atoms (4-F: **2m**, 4-Cl: **2n**, and 3,4-difluoro: **2t**), respectively, at different positions on the aromatic ring showed a good reactivity with the said reactants and afforded the corresponding products **11b**–**h** that ranged from 90 to 96% (Table 7, entries 2–8). Further, the use of pyridine-2-aldehyde (**2u**) resulted in a good isolated yield of **11i** (93%, Table 7, entry 9). In a similar way, the reaction of benzaldehyde (**2a**) with malononitrile (**8**) and thiophenol (**10**) gave the product **11j** in 98% yield (Table 7, entry 10). Benzaldehyde derivatives bearing various functional groups, such as electron-donating groups (4-Me: **2b**, 4-OMe: **2e**, and 3,4,5-trimethoxy: **2g**), an electron-accepting group (4-NO_2_: **2l**), and halogen atoms (4-F: **2m**, 4-Cl: **2n**, and 3-Br: **2s**), respectively, at different positions on the aromatic ring displayed a good reactivity with malononitrile (**8**) and thiophenol (**10**) to give the corresponding products (**11k**–**q**) in good yields, ranging from 94 to 98% (Table 7, entries 11–17). Pyridine-2-aldehyde (**2u**) also provided the product **11r** in a good yield (94%, Table 7, entry 18). It was observed from the above results that all reactions proceeded well irrespective of the substituents present on the (hetero)aromatic aldehyde and afforded the highly substituted pyridines **11** in good isolated yields that ranged from 90 to 98%. Most of the synthesized compounds are known and were identified easily by comparison of the melting point and spectroscopic data with those reported.

**Table 7:** NDL-catalyzed synthesis of 2-amino-4-(hetero)aryl-3,5-dicarbonitrile-6-sulfanylpyridines **11**.^a^

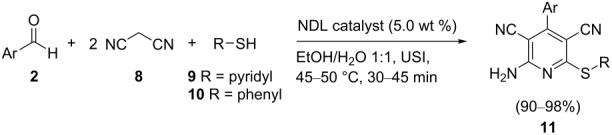							

entry	Ar	R	product	*t* (min)	yield^b^ (%)	mp (°C)	

						found	reported

1	phenyl: **2a**	pyridyl **9**	**11a**	30	96	222–223	224–227 [70]
2	4-methoxyphenyl: **2e**	pyridyl **9**	**11b**	35	96	248–249	250–253 [70]
3	3,4,5-trimethoxyphenyl: **2g**	pyridyl **9**	**11c**	40	92	267–269	265–268 [70]
4	3-hydroxyphenyl: **2r**	pyridyl **9**	**11d**	35	94	223–224	222–226 [70]
5	4-nitrophenyl: **2l**	pyridyl **9**	**11e**	32	96	241–243	245–248 [70]
6	4-fluorophenyl: **2m**	pyridyl: **9**	**11f**	32	95	248–250	246–249 [70]
7	4-bromophenyl: **2o**	pyridyl: **9**	**11g**	30	94	257–258	260–263 [70]
8	3,4-difluorophenyl: **2t**	pyridyl: **9**	**11h**	37	90	252–253	251–254 [70]
9	pyridyl: **2u**	pyridyl: **9**	**11i**	45	93	230–231	233–235 [70]
10	phenyl: **2a**	phenyl: **10**	**11j**	30	98	210–212	215–216 [63]
11	4-methylphenyl: **2b**	phenyl: **10**	**11k**	30	98	206–207	208–210 [69]
12	4-methoxyphenyl: **2e**	phenyl: **10**	**11l**	35	97	234–235	236–238 [64]
13	3,4,5-trimethoxyphenyl: **2g**	phenyl: **10**	**11m**	38	94	240–241	238–239 [63]
14	4-nitrophenyl: **2l**	phenyl: **10**	**11n**	30	95	280–282	286–287 [63]
15	4-fluorophenyl: **2m**	phenyl: **10**	**11o**	30	96	127–128	224–225 [69]
16	4-chlorophenyl: **2n**	phenyl: **10**	**11p**	30	96	220–221	222–223 [69]
17	3-bromophenyl: **2s**	phenyl: **10**	**11q**	34	94	250–253	256–258 [65]
18	pyridyl: **2u**	phenyl: **10**	**11r**	42	94	300–302	305–306 [63]

^a^Reaction conditions: aldehyde (**2**, 1.0 mmol), malononitrile (**8**, 2.0 mmol), thiol **9** or **10** (1.0 mmol), NDL (5.0 wt %), EtOH/H_2_O 1:1 (3.0 mL), USI at 45**–**50 °C. ^b^Isolated yield.

### Evaluation of the green chemistry metrics for the synthesis of benzimidazoles **3**, dihydropyrimidinones **7**, and highly functionalized pyridines **11**

In order to evaluate the “greenness” of the proposed methodologies, the green chemistry metrics, such as the atom economy (AE), E-factor, process mass intensity (PMI), Curzon’s reaction mass efficiency (RME), and generalized or global reaction mass efficiency (gRME) were evaluated by adopting established standard empirical formulae [88–89]. The obtained results are summarized in Tables 8–10. This study revealed that the reactions displayed a good to excellent AE (88–95%) and Curzon’s RME (78–93%) as well as a low to moderate E-factor (26.202–50.760) and PMI (27.202–51.760). The detailed calculations of the green chemistry metrics (AE, E-factor, PMI, Curzon’s RME, and gRME) for the synthesis of the compounds **3a**, **7a**, and **11a** (Table 8, entry 1, Table 9, entry 1, and Table 10, entry 1) are presented in Supporting Information File 1 (see Reaction-S1–Reaction-S3).

**Table 8:** Green chemistry metrics for the synthesis of 2-aryl-1-arylmethyl-1*H*-benzo[*d*]imidazoles **3**.

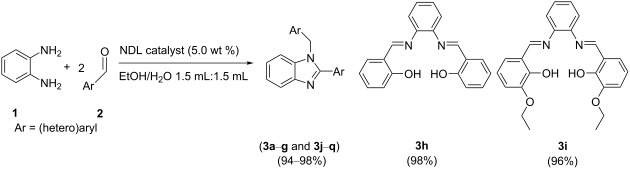							

entry	Ar	product	AE^a^ (%)	E-factor^b^	PMI^c^	Curzon’s RME^d^ (%)	gRME^e^ (%)

1	phenyl: **2a**	**3a**	89	40.864	41.864	87	2.4
2	4-methylphenyl: **2b**	**3b**	90	37.261	38.261	88	2.6
3	4-*tert*-butylphenyl: **2c**	**3c**	92	30.614	31.614	86	3.2
4	2,4-dimethylphenyl: **2d**	**3d**	90	35.044	36.044	86	2.8
5	4-methoxyphenyl: **2e**	**3e**	91	33.837	34.837	89	2.9
6	3,4-dimethoxyphenyl: **2f**	**3f**	92	29.729	30.729	87	3.3
7	3,4,5-trimethoxyphenyl: **2g**	**3g**	93	26.202	27.202	87	3.7
8	2-hydroxyphenyl: **2h**	**3h**	90	36.781	37.781	88	2.6
9	2-hydroxy-3-ethoxyphenyl: **2i**	**3i**	92	29.412	30.412	88	3.3
10	4-hydroxy-3-methoxyphenyl: **2j**	**3j**	91	31.609	32.609	88	3.1
11	4-hydroxy-3-ethoxyphenyl: **2k**	**3k**	92	29.102	30.102	89	3.3
12	4-nitrophenyl: **2l**	**3l**	91	31.017	32.017	89	3.1
13	4-fluorophenyl: **2m**	**3m**	90	36.312	37.312	88	2.7
14	4-chlorophenyl: **2n**	**3n**	91	33.052	34.052	89	2.9
15	4-bromophenyl: **2o**	**3o**	93	26.920	27.920	89	3.6
16	2-furanyl: **2p**	**3p**	88	45.454	46.454	84	2.2
17	2-thienyl: **2q**	**3q**	89	40.169	41.169	85	2.4

^a^AE = 100⋅(GMW of the product/sum of the GMWs of the reactants); GMW = gram molecular weight. ^b^E-factor = total input mass (^m^inputs)^f^ − mass of the target product (^m^**3**) − mass of the recovered materials/^m^**3**. ^c^PMI = (^m^inputs − mass of the recovered materials)/^m^**3** or 1 + E-factor. ^d^Curzon’s RME = ^m^**3**/ ^m^**1** + ^m^**2** or yield × AE × 1/stoichiometric factor (SF); SF = 1. ^e^gRME = 100⋅(^m^**3**/(^m^inputs − mass of the recovered materials)) or 100⋅(1/(1 + E-factor)). ^f^Total input mass, including water (^m^inputs) = ^m^**1** + ^m^**2** + ^m^solvent (S) + ^m^catalyst (C) + ^m^work-up materials (WPM) + ^m^purification materials (PM).

**Table 9:** Green chemistry metrics for the synthesis of dihydropyrimidinones/-thiones **7**.

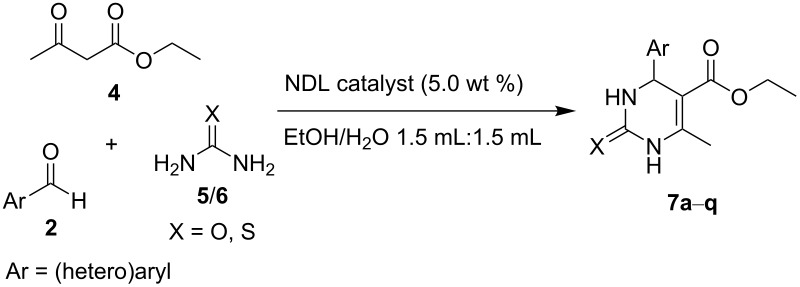								

entry	reactants		product	AE (%)	E-factor^a^	PMI^b^	Curzon’s RME^c^ (%)	gRME^d^ (%)

	Ar	**5**/**6**						

1	phenyl: **2a**	**5**	**7a**	88	45.254	46.254	85	2.2
2	4-methylphenyl: **2b**	**5**	**7b**	88	43.373	44.373	84	2.3
3	4-methoxyphenyl: **2e**	**5**	**7c**	89	41.036	42.036	85	2.4
4	3,4-dimethoxyphenyl: **2f**	**5**	**7d**	90	37.924	38.924	85	2.6
5	3-hydroxyphenyl: **2r**	**5**	**7e**	89	43.550	44.550	85	2.2
6	2-hydroxyphenyl: **2h**	**5**	**7f**	89	44.953	45.953	82	2.2
7	4-nitrophenyl: **2l**	**5**	**7g**	89	39.770	40.770	84	2.5
8	4-fluorophenyl: **2m**	**5**	**7h**	89	43.220	44.220	85	2.3
9	4-chlorophenyl: **2n**	**5**	**7i**	89	40.311	41.311	85	2.4
10	3-bromophenyl: **2s**	**5**	**7j**	90	36.254	37.254	84	2.7
11	2-furanyl: **2p**	**5**	**7k**	87	50.760	51.760	78	1.9
12	2-thienyl: **2q**	**5**	**7l**	88	46.600	47.600	81	2.1
13	phenyl: **2a**	**6**	**7m**	89	43.045	44.045	85	2.3
14	4-methylphenyl: **2b**	**6**	**7n**	89	41.341	41.341	85	2.4
15	4-methoxyphenyl: **2e**	**6**	**7o**	90	39.213	40.213	86	2.5
16	4-nitrophenyl: **2l**	**6**	**7p**	90	37.978	38.978	85	2.6
17	4-chlorophenyl: **2n**	**6**	**7q**	90	38.685	39.685	86	2.5

^a^E-factor = ^m^inputs^e^ − mass of the target product (^m^**7**) − mass of the recovered materials/^m^**7**. ^b^PMI = (^m^inputs − mass of the recovered materials)/^m^**7** or 1 + E-factor. ^c^Curzon’s RME = ^m^**7**/^m^**2** + ^m^ **4** + ^m^**5/6** or yield × AE × 1/SF; SF = 1. ^d^gRME = 100⋅(^m^**7**/(^m^inputs − mass of the recovered materials)) or 100⋅(1/(1 + E-factor)). ^em^inputs = ^m^**2** + ^m^**4** + ^m^**5**/**6** + ^m^S + ^m^C + ^m^WPM + ^m^purification materials (PM).

**Table 10:** Green chemistry metrics for the synthesis of 2-amino-4-(hetero)aryl-3,5-dicarbonitrile-6-sulfanylpyridines **11**.

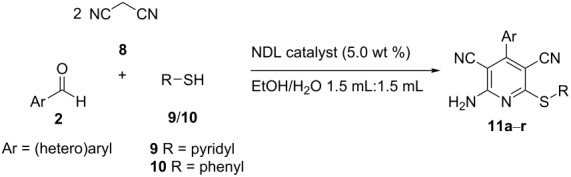								

entry	reactants		product	AE (%)	E-factor^a^	PMI^b^	Curzon’s RME^c^ (%)	gRME^d^ (%)

	Ar	R						

1	phenyl: **2a**	pyridyl: **9**	**11a**	94	36.054	37.054	90	2.7
2	4-methoxyphenyl: **2e**	pyridyl: **9**	**11b**	95	33.026.	34.026	91	2.9
3	3,4,5-trimethoxyphenyl: **2g**	pyridyl: **9**	**11c**	95	29.647	30.647	87	3.3
4	3-hydroxyphenyl: **2r**	pyridyl: **9**	**11d**	95	35.188	36.188	89	2.8
5	4-nitrophenyl: **2l**	pyridyl: **9**	**11e**	95	31.741	32.741	91	3.1
6	4-fluorophenyl: **2m**	pyridyl: **9**	**11f**	95	34.356	35.356	90	2.8
7	4-bromophenyl: **2o**	pyridyl: **9**	**11g**	95	29.698	30.698	89	3.3
8	3,4-difluorophenyl: **2t**	pyridyl: **9**	**11h**	95	34.699	35.699	86	2.8
9	pyridyl: **2u**	pyridyl: **9**	**11i**	94	37.143	38.143	87	2.6
10	Phenyl: **2a**	phenyl: **10**	**11j**	94	35.474	36.474	92	2.7
11	4-methylphenyl: **2b**	phenyl: **10**	**11k**	95	33.991	34.991	93	2.9
12	4-methoxyphenyl: **2e**	phenyl: **10**	**11l**	95	32.827	33.827	92	3.0
13	3,4,5-trimethoxyphenyl: **2g**	phenyl: **10**	**11m**	95	29.020	30.020	89	3.3
14	4-nitrophenyl: **2l**	phenyl: **10**	**11n**	95	32.021	33.021	90	3.0
15	4-fluorophenyl: **2m**	phenyl: **10**	**11o**	95	34.319	35.319	91	2.8
16	4-chlorophenyl: **2n**	phenyl: **10**	**11p**	95	32.744	33.744	91	3.0
17	3-bromophenyl: **2s**	phenyl: **10**	**11q**	95	29.775	30.775	89	3.2
18	pyridyl: **2u**	phenyl: **10**	**11r**	94	36.893	37.893	88	2.6

^a^E-factor = ^m^inputs^f^ − mass of the target product (^m^**11**) − mass of the recovered materials/^m^**11**. ^b^PMI = (^m^inputs − mass of the recovered materials)/^m^**11** or 1 + E-factor. ^c^Curzon’s RME = ^m^**11**/ ^m^**2** + ^m^**8** + ^m^**9/10** or yield × AE × 1/SF; SF = 1. ^d^gRME = 100⋅(^m^**11**/(^m^inputs − mass of the recovered materials)) or 100⋅(1/(1 + E-factor)). ^em^inputs = ^m^**2** + ^m^**8** + ^m^**9/10** + ^m^S + ^m^C + ^m^WPM + ^m^PM.

### Catalyst reusability experiments

Catalyst reusability tests were performed showcasing the synthesis of the compounds **3k**, **7a**, and **11e** under the optimized reaction conditions.

#### Catalyst reusability experiments in the synthesis of compounds **3k**, **7a**, and **11e**

The catalyst was tested for reusability in the preparation of **3k** using *o*-phenylenediamine (**1**) and 3-ethoxy-4-hydroxybenzaldehyde (**2k**) under USI for 10 min. After completion of the first reaction cycle, the reaction mass was allowed to cool to rt, and ethyl acetate (4.0 mL) was added. Then, the catalyst was separated by vacuum filtration, washed with ethyl acetate (1.0 mL), dried under vacuum, and reused in the next cycles. The study revealed that the obtained yields of the product, **3k** were 98, 98, 97, 97, 96, 97, and 98% for the first, second, third, fourth, fifth, sixth, and seventh cycle, respectively. Catalyst reusability tests were then conducted for the synthesis of compound **7a** using benzaldehyde (**2a**), ethyl acetoacetate (**4**), and urea (**5**) under USI for 15 min and for **11e** using 4-nitrobenzaldehyde (**2l**), malononitrile (**8**), and 2-mercaptopyridine (**9**) under USI for 32 min by following the same procedure as adopted for **3k**. The yields obtained for the compounds were 97, 97, 97, 96, 97, 97, and 97% for **7a** as well as 96, 96, 96, 97, 97, 97, and 98% for **11e** for the first, second, third, fourth, fifth, sixth, and seventh cycle, respectively. From this study, it was noticed that the catalyst could successfully be reused (at least) 7 times in the synthesis of the compounds **3k**, **7a**, and **11e** without a significant loss of the catalytic activity.

#### Effect of ultrasonication on the structure of the catalyst

**Figure 8:**
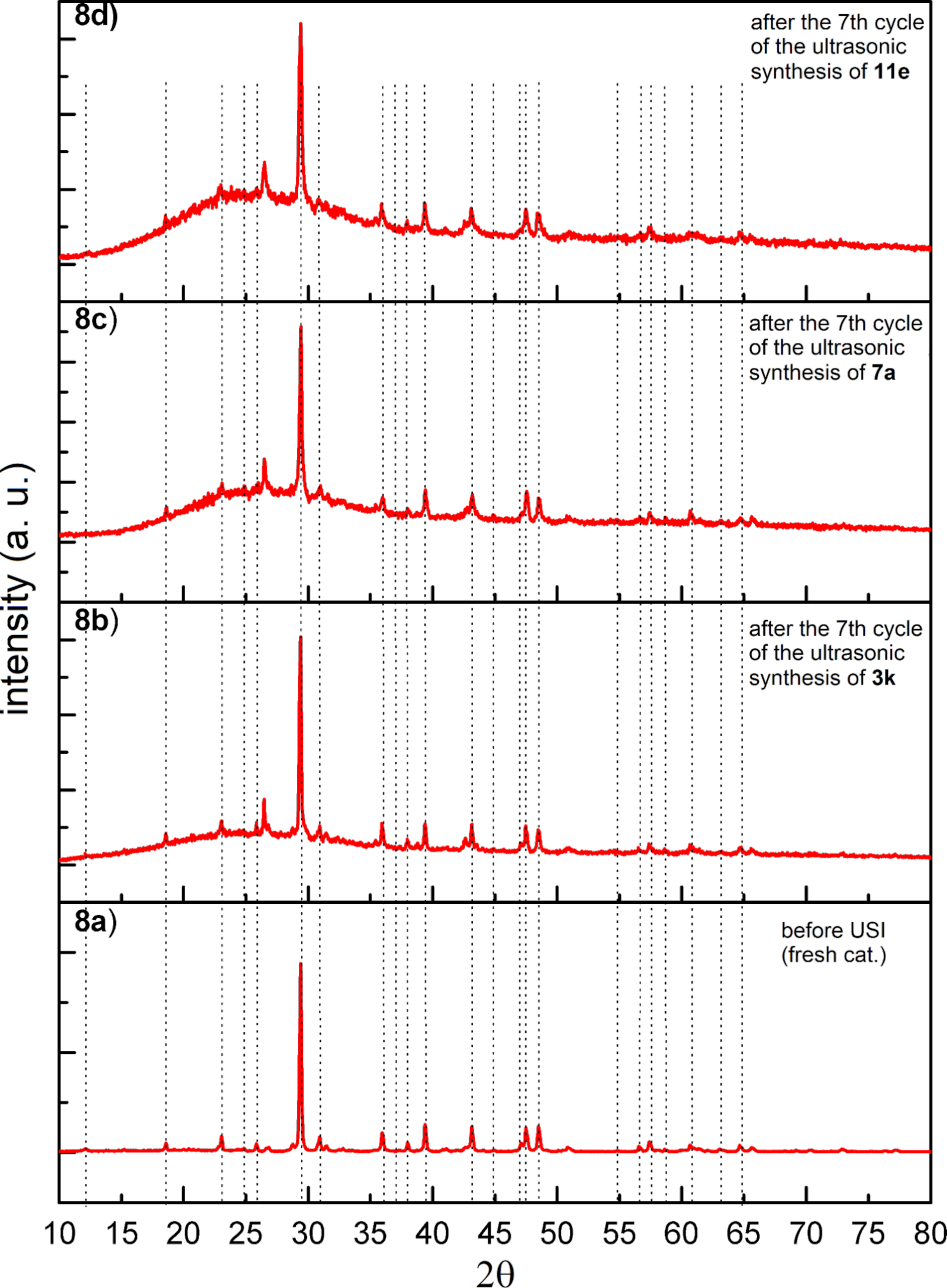
XRD pattern of a) the fresh NDL catalyst; b) the recovered NDL catalyst after the 7th cycle of the ultrasonic synthesis of **3k**; c) the recovered NDL catalyst after the 7th cycle of the ultrasonic synthesis of **7a**; and d) the recovered NDL catalyst after the 7th cycle of the ultrasonic synthesis of **11e**.

The recovered catalyst after the 7th cycle of each synthesis was characterized by XRD to study the structural changes due to ultrasonication. As can be seen in Figure 8, the diffraction peak positions of the catalyst recovered after the synthesis of the compounds **3k**, **7a**, and **11e** (Figure 8b–d), respectively, remained the same as compared to the fresh catalyst (Figure 8a). It was also noticed that the broadening in the XRD pattern of the recovered catalyst had increased with an increase of the ultrasonication time. This clearly indicated that the amorphization of the recovered catalyst was enhanced by increasing the ultrasonication time.

## Conclusion

An environmentally benign NDL catalyst was characterized and utilized as a heterogeneous catalyst for the synthesis of 2-aryl-1-arylmethyl-1*H*-benzo[*d*]imidazoles, dihydropyrimidinones/ -thiones, and 2-amino-4-(hetero)aryl-3,5-dicarbonitrile-6-sulfanylpyridines in a mixture of ethanol and H_2_O 1:1 under ultrasound irradiation. Notable advantages of this methodology include the clean reaction profile, broad substrate scope, simplicity of the process and handling, low catalyst loading, and the easy and quick isolation of the products in good to excellent yield. Besides, the products obtained were in an adequate purity without the need for chromatographic separation, and the catalyst was reused 7 times without a significant loss of the catalytic activity. Hence, the catalyst is a greener alternative for the synthesis of 1,2-disubstituted benzimidazoles, dihydropyrimidinones/-thiones, and highly substituted pyridines when compared to the existing reported catalysts. Further, the expansion of the catalyst scope and the generality for the synthesis of other privileged nitrogen- and sulfur-based heterocycles is under progress in our laboratory.

## Experimental

See Supporting Information File 1 for full experimental data of compounds **3**, **7**, and **11**.

## Supporting Information

File 1 Experimental procedures, characterization data, and copies of the ^1^H and ^13^C NMR, mass, and HRMS spectra of **3**, **7**, and **11**.
