# Supplementary material for: Natural dolomitic limestone-catalyzed synthesis of benzimidazoles, dihydropyrimidinones, and highly substituted pyridines under ultrasound irradiation
Source: Beilstein J Org Chem. 2020 Aug 3;16:1881–900. doi: 10.3762/bjoc.16.156 (PMC7404249; doi:10.3762/bjoc.16.156)
Supplement: File 1 — Experimental procedures, characterization data, and copies of the 1H and 13C NMR, mass, and HRMS spectra of 3, 7, and 11. [file Beilstein_J_Org_Chem-16-1881-s001.pdf]

BEILSTEIN JOURNAL OF ORGANIC CHEMISTRY

Supporting Information

for

Natural dolomitic limestone-catalyzed synthesis of benzimidazoles, dihydropyrimidinones, and highly substituted pyridines under ultrasound irradiation

Kumar Godugu, Venkata Divya Sri Yadala, Mohammad Khaja Mohinuddin Pinjari, Trivikram Reddy Gundala, Lakshmi Reddy Sanapareddy and Chinna Gangi Reddy Nallagondu

Beilstein J. Org. Chem. **2020,** *16,* 1881–1900. doi:10.3762/bjoc.16.156

Experimental procedures, characterization data, and copies of the ^{1}H and ^{13}C NMR, mass, and HRMS spectra of 3, 7, and 11

License and Terms: This is a supporting information file under the terms of the Creative Commons Attribution License (http://creativecommons.org/licenses/by/4.0). Please note that the reuse, redistribution and reproduction in particular requires that the authors and source are credited.
The license is subject to the *Beilstein Journal of Organic Chemistry* terms and conditions: (https://www.beilstein-journals.org/bjoc)

Table of contents

Contents	Page no.
Experimental section	S1–S3
Physical and spectral data as well as copies of the ^{1}H and ^{13}C NMR, mass, and HRMS spectra of **3**, **7**, and **11**	S3–S22
Detailed calculations of green chemistry metrics for the synthesis of compounds **3a**, **7a**, and **11a**	S22–S27

Experimental

Materials and methods

The ^{1}H (300, 400, and 500 MHz) and ^{13}C (75, 100, and 125 MHz) NMR spectroscopic data were recorded on a Varian spectrometer. High-resolution mass spectra were recorded on a Bruker micrOTOF-QII mass spectrometer. Mass spectral data were acquired on an Exactive Orbitrap mass spectrometer (Thermo Scientific, Waltham, MA), equipped with a Thermo Accela 600 quaternary gradient pump, and the MS source was equipped with an electrospray ionization (ESI) probe. XRD patterns were recorded using a Rigaku Miniflex 600 diffractometer. FTIR spectra were recorded on a PerkinElmer FTIR spectrometer (PerkinElmer, Spectrum Two model, Singapore). Raman spectra were recorded on a Lab RAM high-resolution Raman spectrometer (Horiba). Scanning electron microscopy (SEM) images were recorded on a JSM-IT500 scanning electron microscope (JEOL). Ultrasonic irradiation was carried out by using a Bandelin SONOREX RK 510 H (with a frequency of 35 kHz and a power of 160/640 W) ultrasonic bath with built-in heating, 30–80 °C (86–176 °F), thermostatically adjustable. The reaction vessel was placed in the bath containing water. The conventional method was performed by stirring the reactants in the round-bottom flask at 550 rpm in an oil bath at different temperatures. Thin-layer chromatography was performed on 0.25 mm Merck silica gel plates and visualized with UV light. Melting points of the various products obtained were determined and are uncorrected. Chemicals and solvents were purchased from Avra, Acros Organics Ltd., and Merck and were used as received.

General experimental procedure for the synthesis of the 2-aryl-1-arylmethyl-1*H*-benzo[*d*]imidazoles 3

A mixture of *o*-phenylenediamine (**1**, 1.0 mmol), the aromatic/heteroaromatic aldehyde **2** (2.0 mmol), and dolomitic limestone (5.0 wt %) was irradiated by ultrasound in a mixture of ethanol and H_2O (1:1; 1.5 mL:1.5 mL) at 45–50 °C for 10–15 min. The progress of the reaction was monitored by TLC. After completion of the reaction, the reaction mass was allowed to cool to rt, and to it was added ethyl acetate (4.0 mL). Then, the catalyst was separated by filtration under vacuum and washed with ethyl acetate (1.0 mL). The filtrate was concentrated under reduced pressure to obtain the crude product **3**. The formed crude solid **3** was further purified by recrystallization using hot aqueous ethanol (1.5 mL ethanol and 3.0 mL water).

General experimental procedure for the synthesis of the dihydropyrimidinone/-thione derivatives 7

A mixture of the aromatic/heteroaromatic aldehyde **2** (1.0 mmol), ethyl acetoacetate (**4**, 1.0 mmol), urea/thiourea (**5/6**, 1.0 mmol), and dolomitic limestone (5.0 wt %) was subjected to ultrasonication in a mixture of ethanol and H_2O (1:1; 1.5 mL:1.5 mL) at 45–50°C for 10–20 min. The progress of the reaction was monitored by TLC. After completion of the reaction, the reaction mass was allowed to cool to rt, and to it was added ethyl acetate (4.0 mL). Then, the catalyst was separated by filtration under vacuum and washed with ethyl acetate (1.0 mL). The filtrate was concentrated under reduced pressure to obtain the crude product **7**. The formed crude solid **7** was further purified by recrystallization using hot aqueous ethanol (1.5 mL ethanol/3.0 mL water).

General experimental procedure for the synthesis of the 2-amino-4-aryl-3,5-dicarbonitrile-6-sulfanylpyridines (11)

A mixture of the aromatic/heteroaromatic aldehyde **2** (1.0 mmol), malononitrile (**8**, 2.0 mmol), the thiol **9/10** (1.0 mmol), and dolomitic limestone (5.0 wt %) was irradiated by ultrasound in a mixture of ethanol and H_2O (1:1; 1.5 mL:1.5 mL) at 45–50°C for 30–45 min. The progress of the reaction was monitored by TLC. After completion of the reaction,

the reaction mass was allowed to cool to rt, and to it was added ethyl acetate (4.0 mL). Then, the catalyst was separated by filtration under vacuum and washed with ethyl acetate (1.0 mL). The filtrate was concentrated under reduced pressure to obtain the crude product **11**. The formed crude solid **11** was further purified by recrystalization using hot aqueous ethanol (1.5 mL ethanol/3.0 mL water).

Physical and spectral data as well as copies of the ^{1}H and ^{13}C NMR, mass, and HRMS spectra of 3, 7, and 11

1-Benzyl-2-phenyl-1*H*-benzo[*d*]imidazole (3a): White solid, m.p. 128–131 °C. ^{1}H NMR (300 MHz, $CDCl_3$, δ/ppm): 7.88 (d, J = 6.0 Hz, 1H, arom H), 7.69 (dd, J = 6.0 Hz, J = 1.5 Hz, 2H, arom H), 7.49-7.44 (m, 3H, arom H), 7.33-7.20 (m, 6H, arom H), 7.11 (d, J = 6.3 Hz, 2H, arom H), 5.46 (s, 2 H, -C**H**$_2$-).

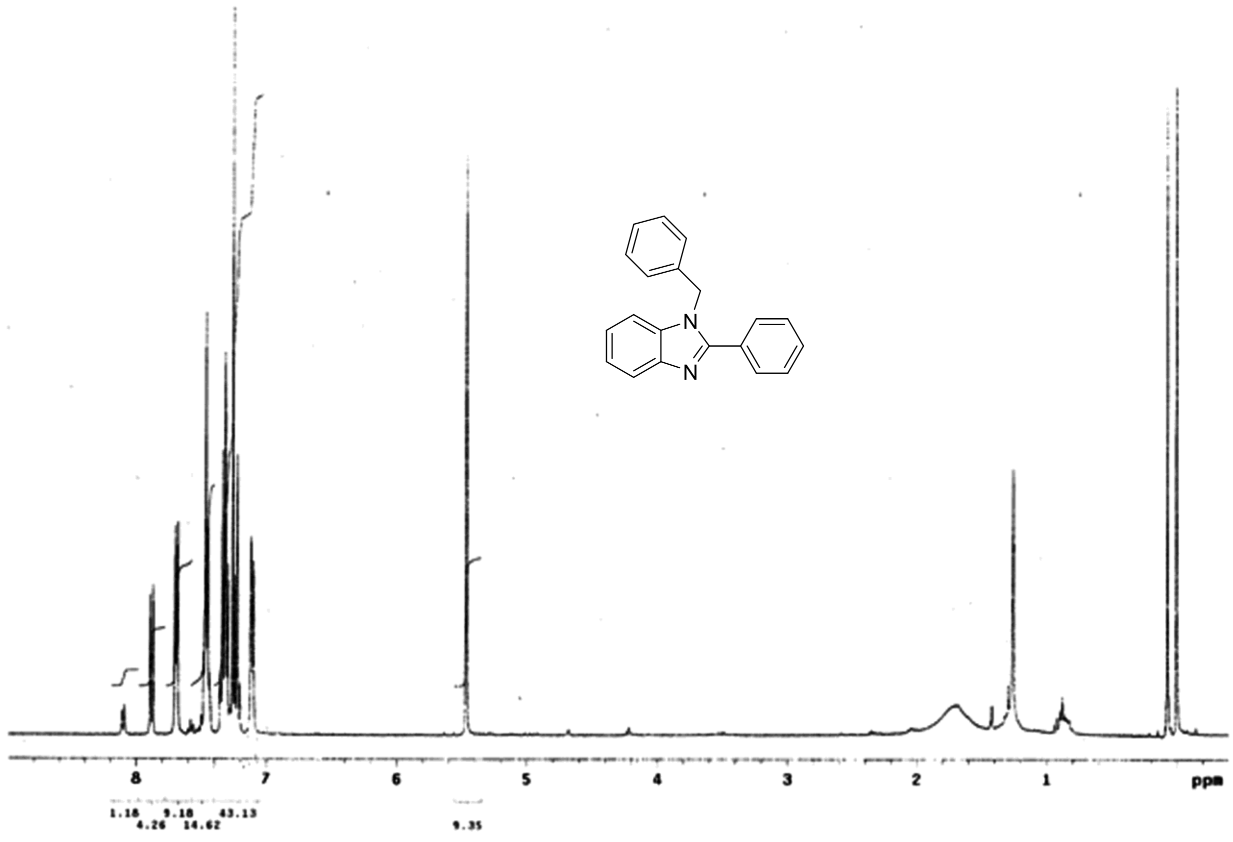

Fig. S1. ^{1}H NMR spectrum of **3a**.

1-(4-Methylbenzyl)-2-(*p*-tolyl)-1*H*-benzo[d]imidazole (3b): White solid, m.p. 127-128 °C. ^{1}H NMR (300 MHz, $CDCl_3$, δ/ppm): 7.85 (d, *J* = 6.0 Hz, 1H, arom H), 7.59 (d, *J* = 6.0 Hz, 2H, arom H), 7.31-7.18 (m, 5H, arom H), 7.13 (d, *J* = 6.0 Hz, 2H, arom H), 7.0 (d, *J* = 6.0 Hz, 2H, arom H), 5.41 (s, 2 H, -C**H**$_2$-), 2.65 (s, 3H, -C**H**$_3$), 2.40 (s, 3H, -C**H**$_3$); MS (ESI): $[M+H]^+$ 313.00

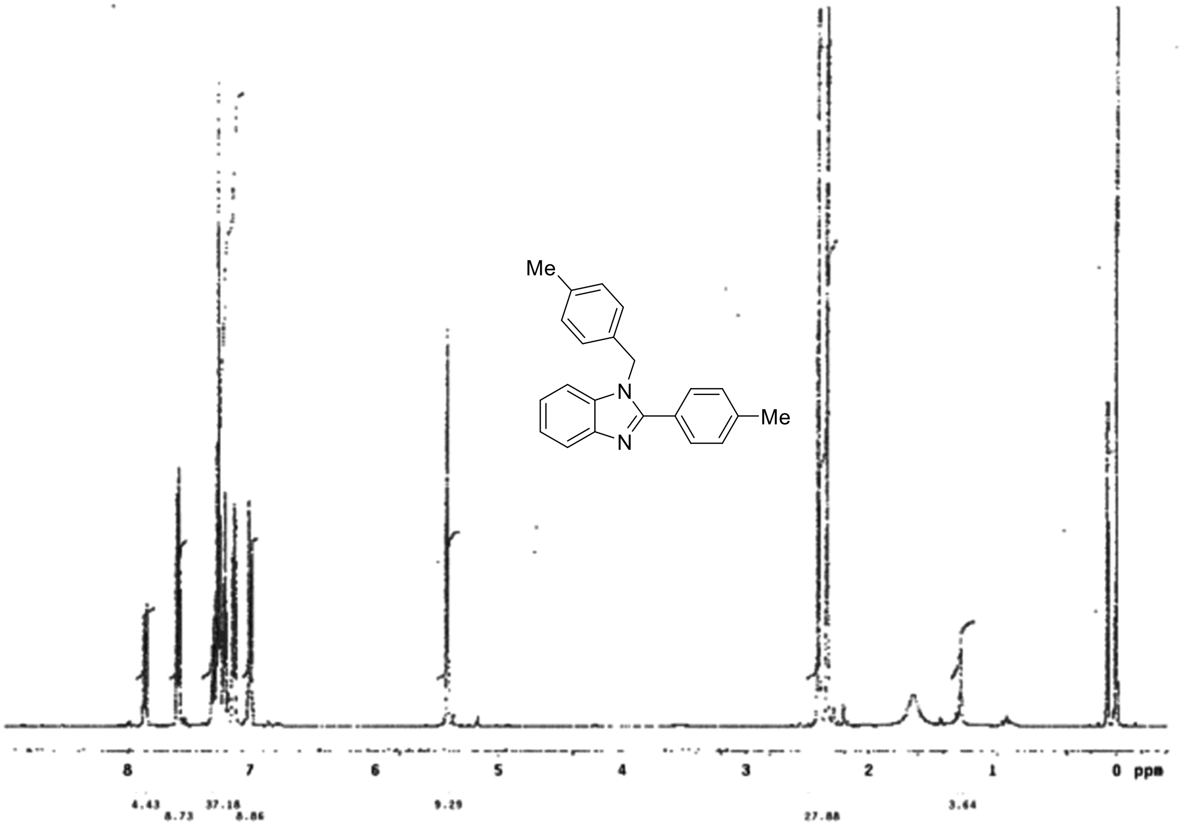

Fig. S2. ^{1}H NMR spectrum of **3b**.

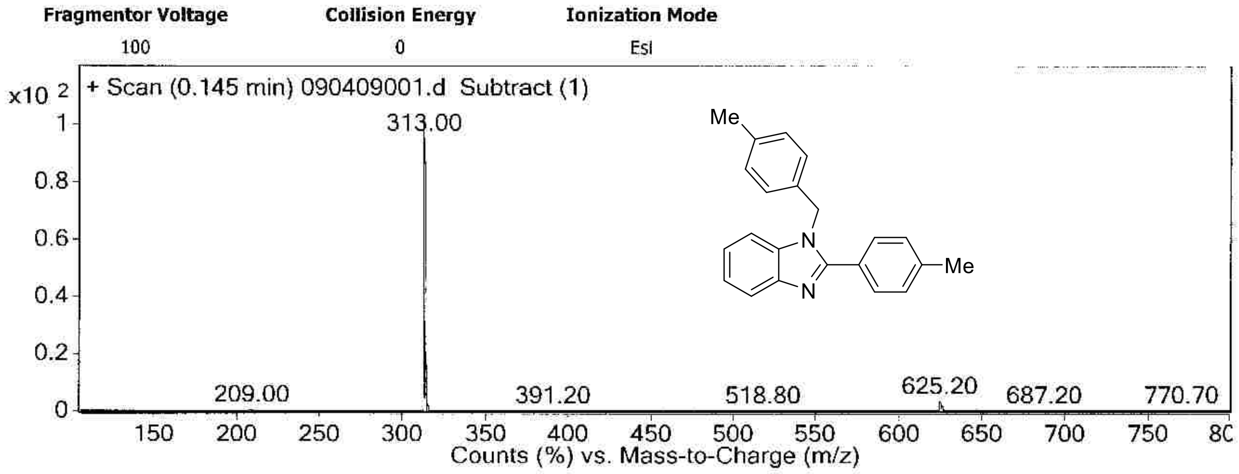

Fig. S3. Mass spectrum of **3b**.

1-(4-(*tert*-Butyl)benzyl)-2-(4-(*tert*-butyl)phenyl)-1*H*-benzo[*d*]imidazole (3c): White solid, m.p. 124-125 °C. ^{1}H NMR (300 MHz, $CDCl_3$, δ/ppm): 7.86 (d, *J* = 6.3 Hz, 1H, arom H), 7.66 (dd, *J* = 4.8 Hz, *J* = 1.2 Hz, 2H, arom H), 7.44(dd, *J* = 6.6 Hz, *J* = 1.5 Hz, 2H, arom H), 7.31-7.20 (m, 5H, arom H), 7.05 (d, *J* = 6.3 Hz, 2H, arom H), 5.45 (s, 2 H, -C**H**$_2$-), 1.34 (s, 9H, -C(C**H**$_3$)$_3$), 1.30 (s, 9H, -C(C**H**$_3$)$_3$); Anal. Calcd. For $C_{28}H_{32}N_2$: C, 84.80; H, 8.13; N, 7.06 Found: C, 84.82; H, 8.15; N, 7.09; MS (ESI): $[M+H]^+$ 397.30

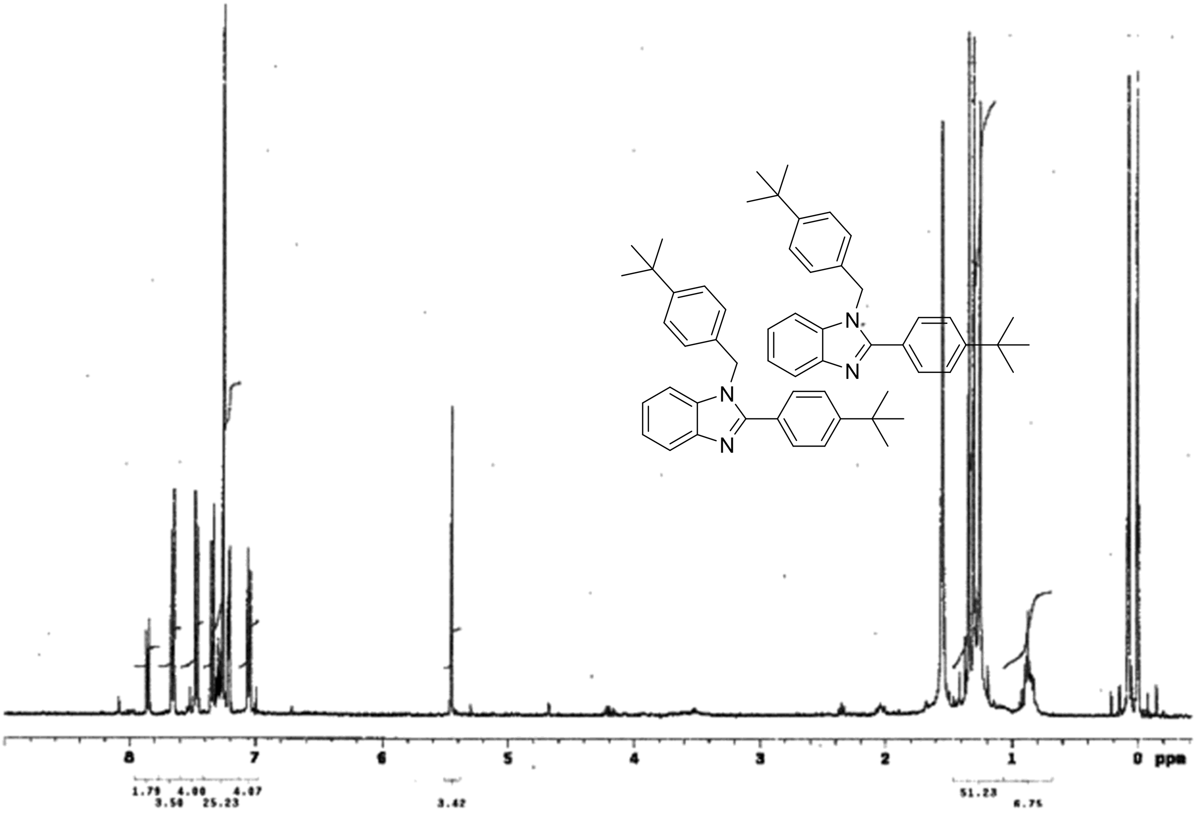

Fig. S4. ^{1}H NMR spectrum of **3c**.

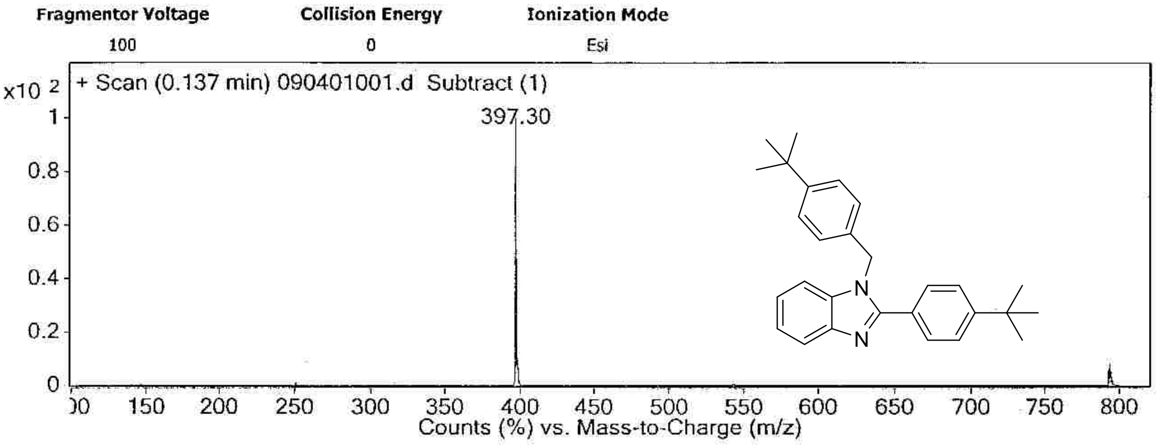

Fig. S5. Mass spectrum of **3c**.

1-(2,4-Dimethylbenzyl)-2-(2,4-dimethylphenyl)-1*H*-benzo[*d*]imidazole (3d): White solid, m.p. 120-122 °C. ^{1}H NMR (300 MHz, $CDCl_3$, δ/ppm): 7.85 (d, *J* = 6.3 Hz, 1H, arom H), 7.31-7.22 (m, 2H, arom H), 7.2-7.15 (m, 4H, arom H), 7.1-6.95 (m, 2H, arom H), 6.84 (d, *J* = 5.7 Hz, 1H, arom H), 6.52 (d, *J* = 5.7 Hz, 1H, arom H), 5.13 (s, 2 H, -C**H$_2$**-), 2.35 (s, 3H, -C**H$_3$**), 2.26(s, 3H, -C**H$_3$**) 2.22 (s, 3H, -C**H$_3$**), 2.13 (s, 3H, -C**H$_3$**); Anal. Calcd. For $C_{24}H_{24}N_2$: C, 84.67; H, 7.11; N, 8.23 Found: C, 84.68; H, 7.15; N, 8.21; MS (ESI): $[M+H]^+$ 341.10

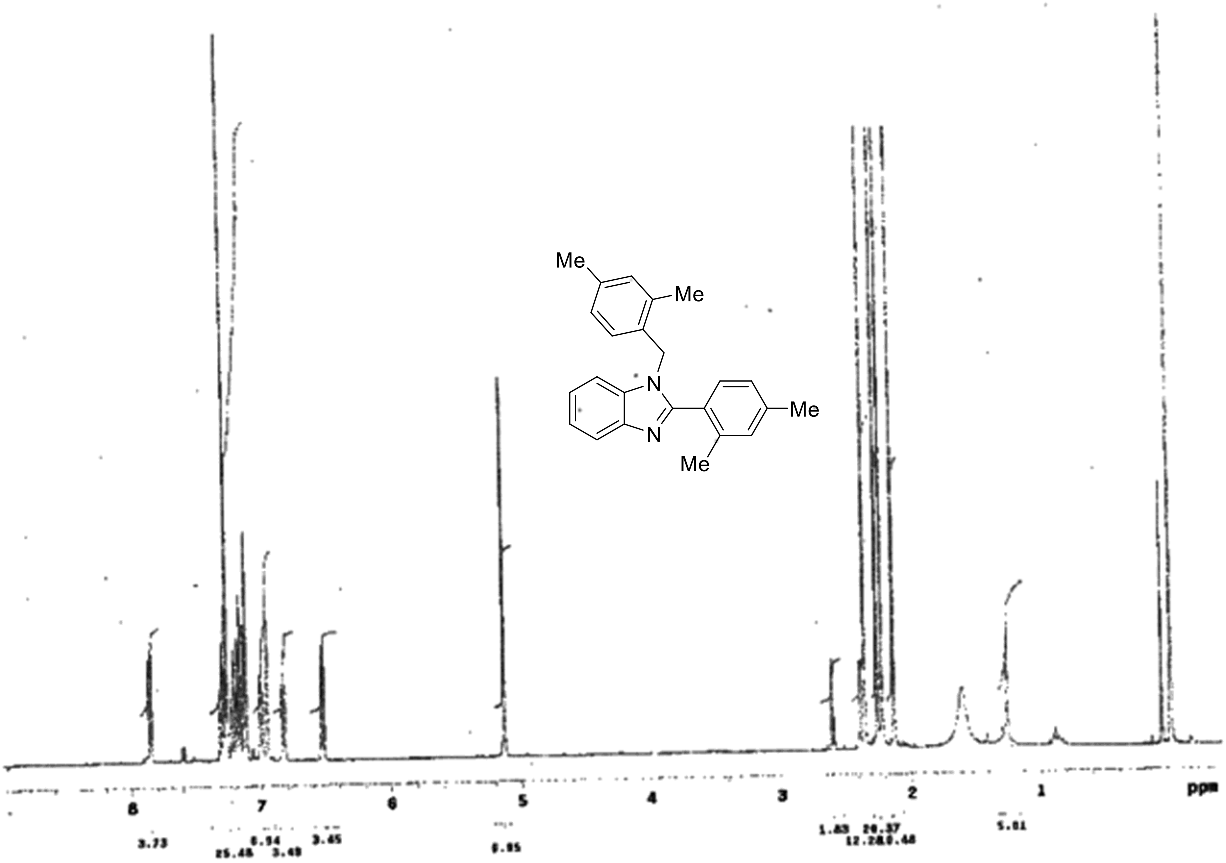

Fig. S6. ^{1}H NMR spectrum of **3d**.

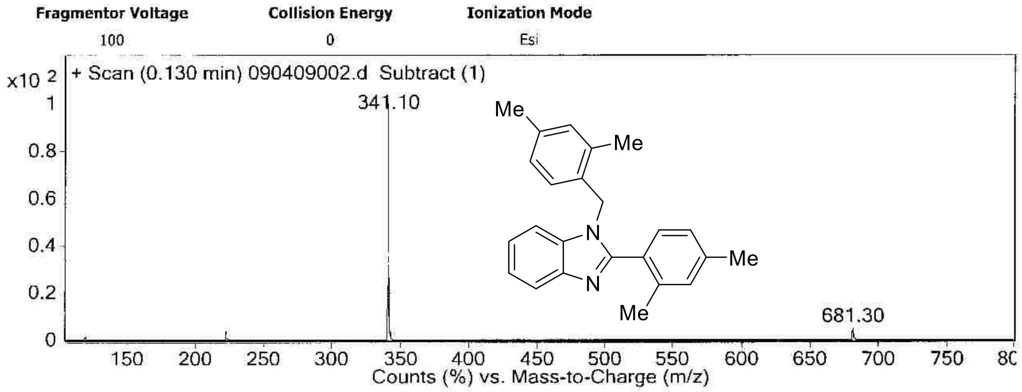

Fig. S7. Mass spectrum of **3d**.

1-(4-Methoxybenzyl)-2-(4-methoxyphenyl)-1*H*-benzo[*d*]imidazole (3e): Brown solid, m.p. 157-159 °C. MS (ESI): $[M+H]^+$ 345.10

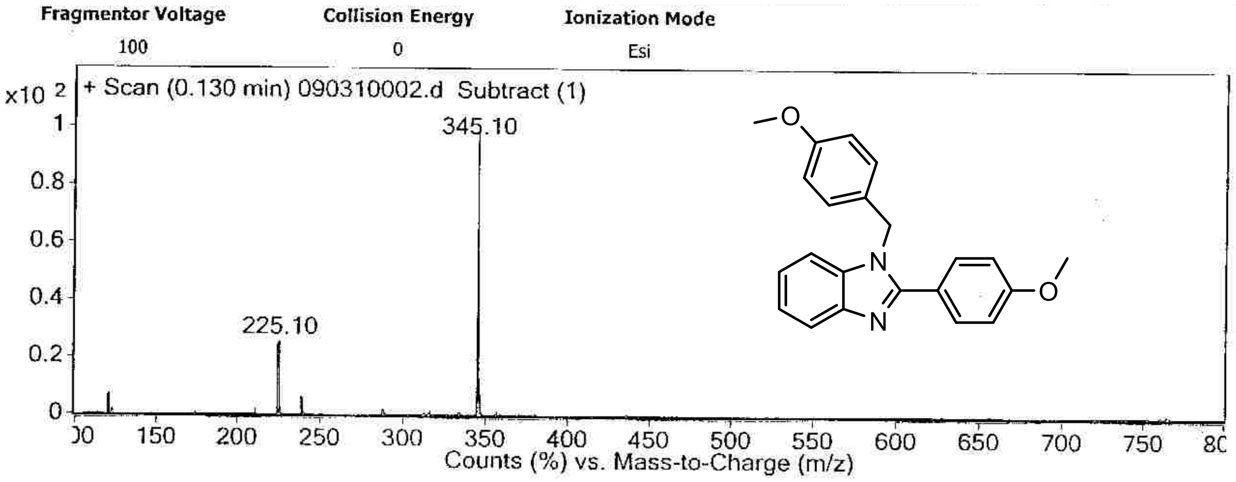

Fig. S8. Mass spectrum of **3e**.

1-(3,4-Dimethoxybenzyl)-2-(3,4-dimethoxyphenyl)-1*H*-benzo[*d*]imidazole (3f): Off white solid, m.p. 167-169 °C. MS (ESI): $[M+H]^+$ 405.20

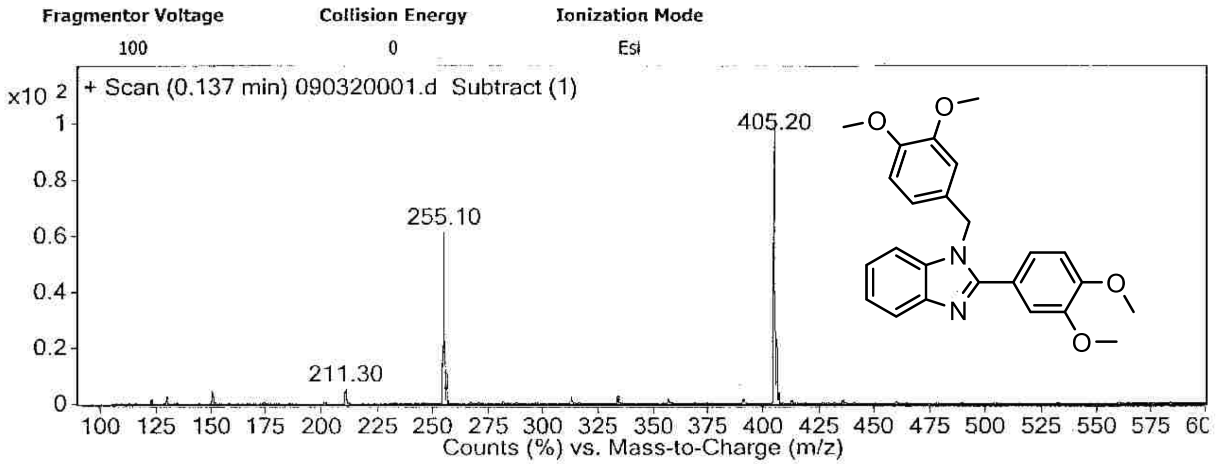

Fig. S9. Mass spectrum of **3f**.

2,2'-((1*E*,1'*E*)-(1,2-Phenylenebis(azanylylidene))bis(methanylylidene))diphenol (bisimine I, 3h): White solid, m.p. 247-248 °C. ^{1}H NMR (500 MHz, $CDCl_3$, δ/ppm): 13.09 (s, 2H,-O**H**), 8.66 (s, 2H, -N**H**), 7.42-7.36 (m, 6H, arom H), 7.27-7.25 (m, 2H, arom H), 7.07 (d, J = 8.5 Hz, 2H, arom H), 6.94 (td, J = 7.5 Hz, J = 1.0 Hz, 2H, arom H). ^{13}C NMR (125 MHz, $CDCl_3$, δ/ppm): 164.52, 160.86, 142.75, 133.92, 132.94, 128.92, 120.24, 119.97, 119.57, 117.16. HRMS (ESI): Anal. Calcd. For $C_{20}H_{16}N_2O_2$: $[M+H]^+$ 317.1290; Found : 317.1285.

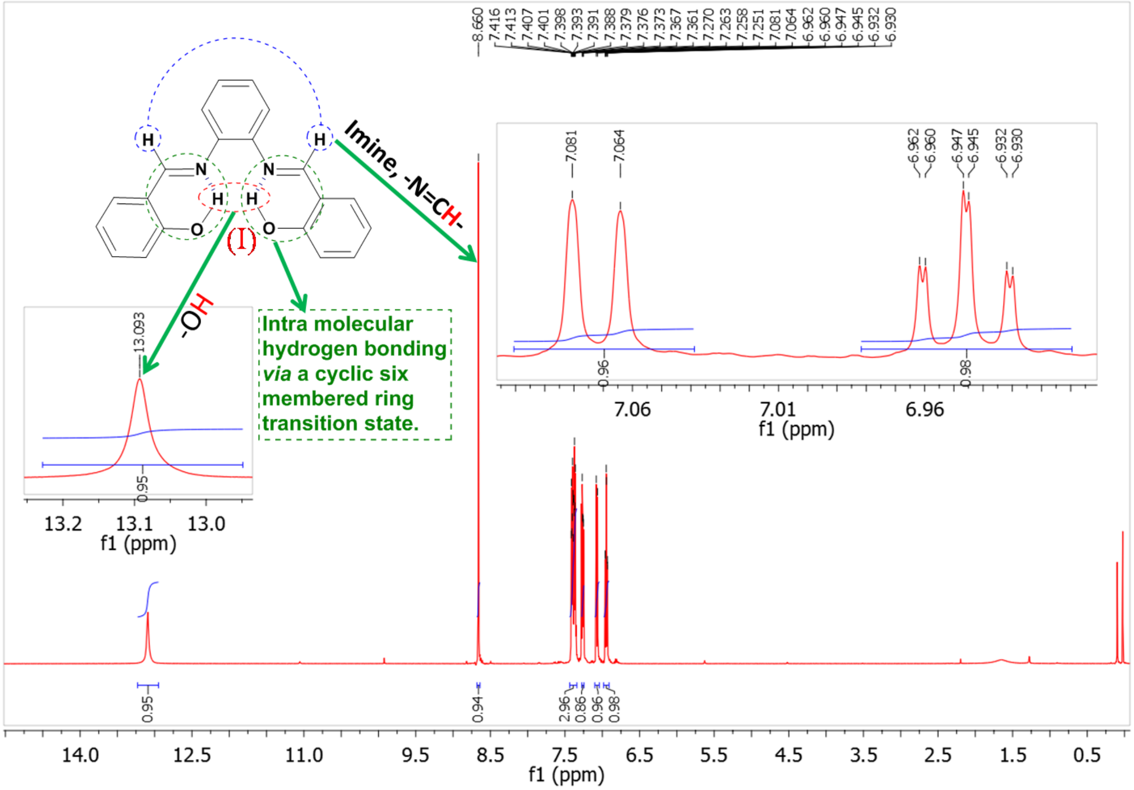

Fig. S10. ^{1}HNMR spectrum of **3h**.

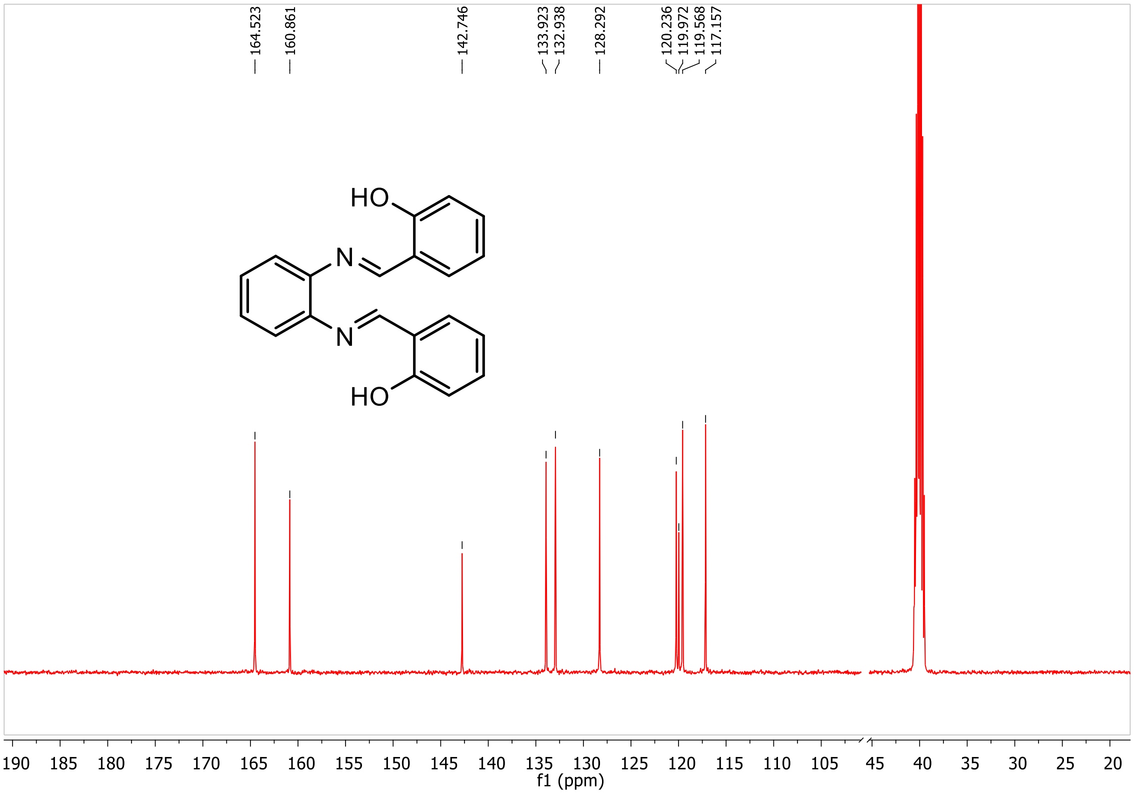

Fig. S11. ^{13}CNMR spectrum of **3h**.

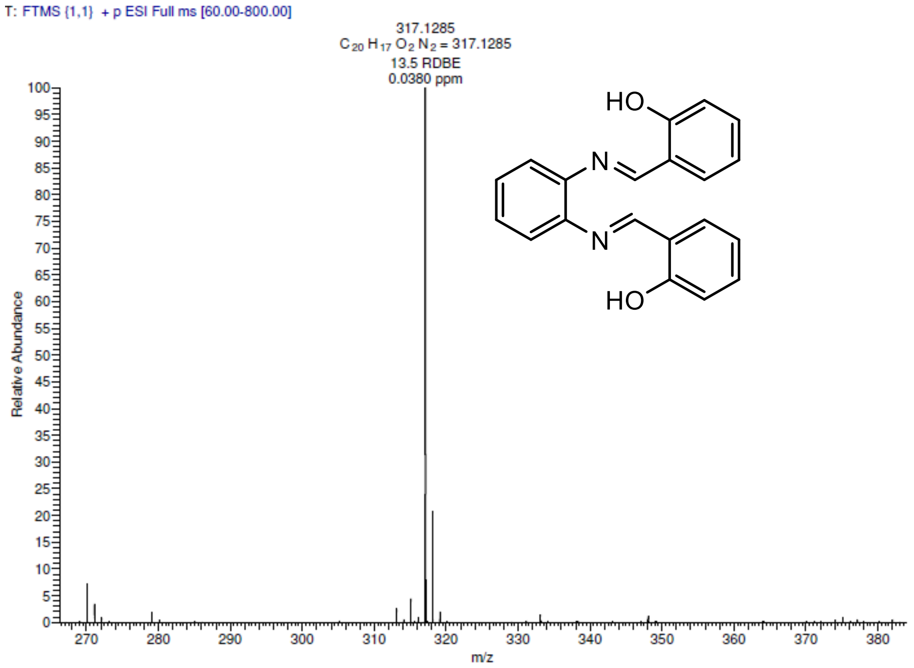

Fig. S12. HRMS spectrum of **3h.**

6,6'-((1*E*,1'*E*)-(1,2-Phenylenebis(azanylylidene))bis(methanylylidene))bis(2-ethoxyphenol) (3i): White solid, m.p. 235-237 °C. ^{1}H NMR (500 MHz, $CDCl_3$, δ/ppm): 13.52 (s, 2H,-O**H**), 8.66 (s, 2H, -N**H**), 7.12 (dd, *J* =7.5 Hz, *J* =1.0 Hz, 1H, arom H), 7.11 (dd, *J* =8.5 Hz, *J* =1.5 Hz, 1H, arom H), 7.10-7.08 (m, 1H, arom H), 7.06 (dd, *J* =7.0 Hz, *J* =1.5 Hz, 2H, arom H), 7.02 (dd, *J* =8.0 Hz, *J* =1.0 Hz, 2H, arom H), 6.91 (t, *J*=8.0 Hz, 2H, arom H), 6.81 (td, *J*=7.0 Hz, *J*=1. Hz, 3H, arom H), 4.17 (q, *J*=7.0 Hz, 4H, -2OC**H**$_2$), 1.53 (t, *J*=7.0 Hz, 6H, -2C**H**$_3$).

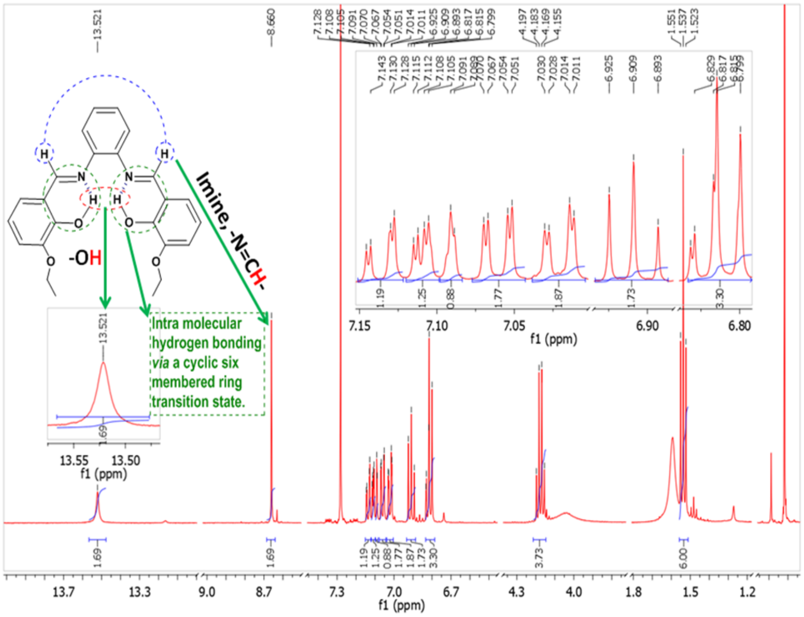

Fig. S13. ^{1}HNMR spectrum of **3i**.

2-thoxy-4-(1-(3-ethoxy-4-hydroxybenzyl)-1H-benzo[d]imidazol-2-yl)phenol (3k): White solid, m.p. 228-230 °C. ^{1}H NMR (500 MHz, $CDCl_3$, *δ*/ppm): 9.77 (s, 1H,-O**H**), 7.79 (d, *J* =8.0 Hz, 1H, arom H), 7.27-7.20 (m, 3H, arom H), 7.12 (dd, *J* =8.0 Hz, *J* =2.0 Hz, 1H, arom H), 6.93 (d, *J* =8.0 Hz, 1H, arom H), 6.83 (d, *J* =8.0 Hz, 1H, arom H), 6.58 (dd, *J* =8.0 Hz, *J* =2.0 Hz, 1H, arom H), 6.54 (d, *J* =2.0 Hz, 1H, arom H), 6.33 (brs, 1H,-O**H**), 5.34 (s, 2H, -C**H**$_2$), 3.98 (q, *J*=7.0 Hz, 2H, -OC**H**$_2$), 3.91 (q, *J*=7.0 Hz, 2H, -OC**H**$_2$), 1.36 (t, *J*=7.0 Hz, 3H, -C**H**$_3$), 1.34 (t, *J*=7.0 Hz, 3H, -C**H**$_3$). HRMS (ESI) Anal. Calcd. For $C_{24}H_{24}N_2O_4$: $[M+H]^+$ 405.1809, found: 405.1810.

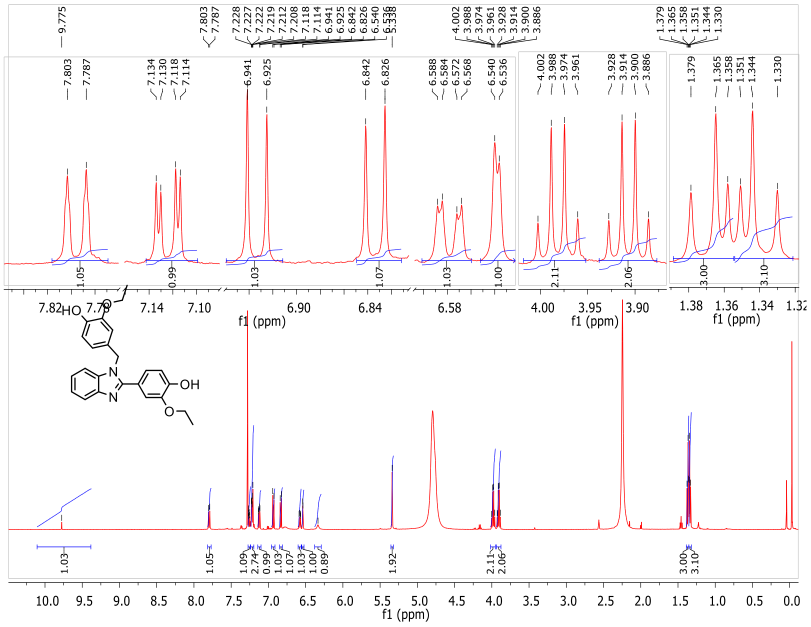

Fig. 14. ^{1}H ^{1}HNMR spectrum of **3k**.

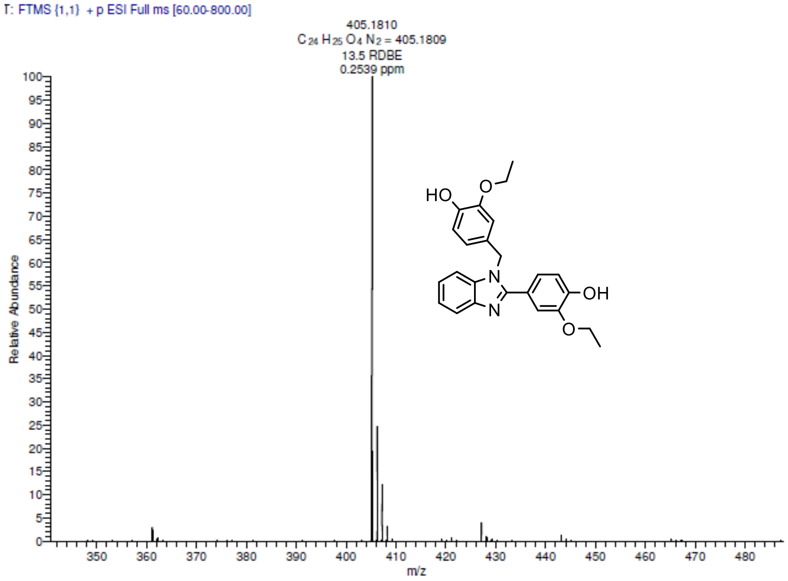

Fig. S15. HRMS spectrum of **3k**.

1-(4-Nitrobenzyl)-2-(4-nitrophenyl)-1*H*-benzimidazole (3l): Yellow solid, m.p. 190-192 °C. MS (ESI): $[M+H]^+$ 375.10

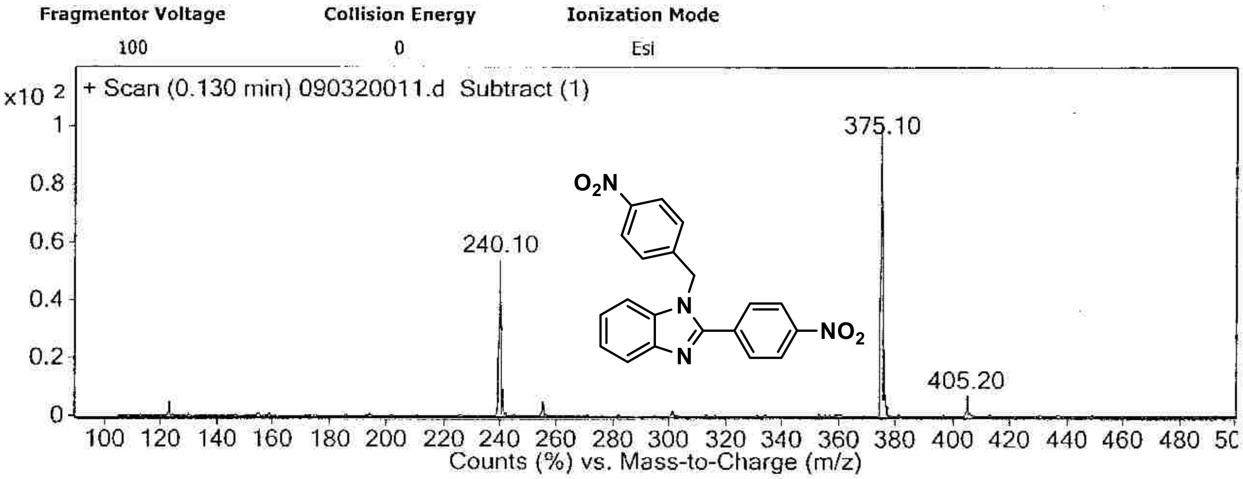

Fig. S16. Mass spectrum of **3l**.

1-(4-Chlorobenzyl)-2-(4-chlorophenyl)-1*H*-benzimidazole (3n): Yellow solid, m.p. 138-140°C. ^{1}H NMR (300 MHz, $CDCl_3$, δ/ppm): 7.88 (d, *J* = 6.3 Hz, 1H, arom H), 7.60 (dd, *J* = 4.8 Hz, *J* = 1.5 Hz, 2H, arom H), 7.44(d, *J* = 6.0 Hz, 2H, arom H), 7.36-7.20 (m, 5H, arom H), 7.02 (d, *J* = 6.3 Hz, 2H, arom H), 5.40 (s, 2 H, -C**H**$_2$).

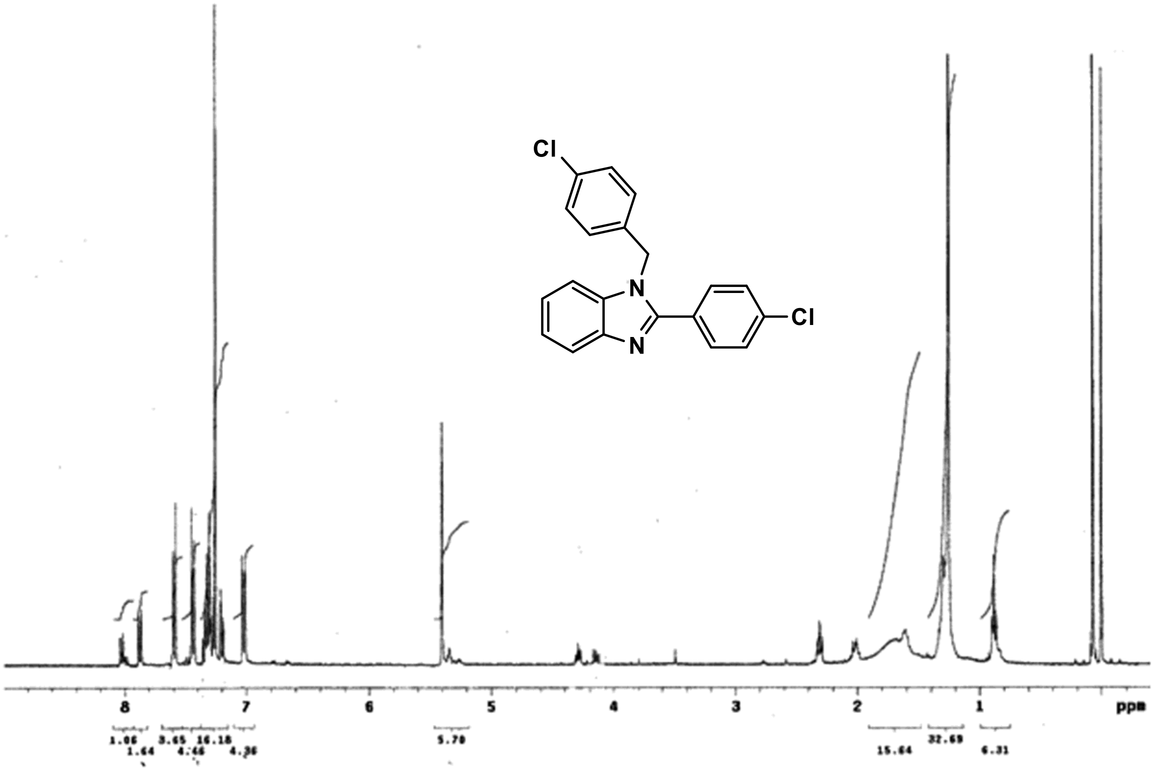

Fig. S17. ^{1}HNMR spectrum of **3n**.

2-(Thiophen-2-yl)-1-(thiophen-2-ylmethyl)-1*H*-benzo[d]imidazole (3q): White solid, m.p. 149-150°C; ^{1}H NMR (300 MHz, $CDCl_3$, δ/ppm): 7.83 (d, *J* = 6.6 Hz, 1H, arom H); 7.53 (d, *J* = 6.3, 1H, arom H), 7.47 (d, *J* = 6.0, 1H, arom H), 7.40(d, *J* = 6.6 Hz, 1H, arom H), 7.32-7.23 (m, 3H, arom H), 7.17 (d, *J* = 6.0 Hz, 1H), 6.90 (dd, *J* = 8.0 and 2.0 Hz, 1H, arom H), 6.87 (d, *J* = 6.2 Hz, 1H, arom H), 5.72 (s, 2H, -C**H2**), MS (ESI): $[M+H]^+$ 297.00

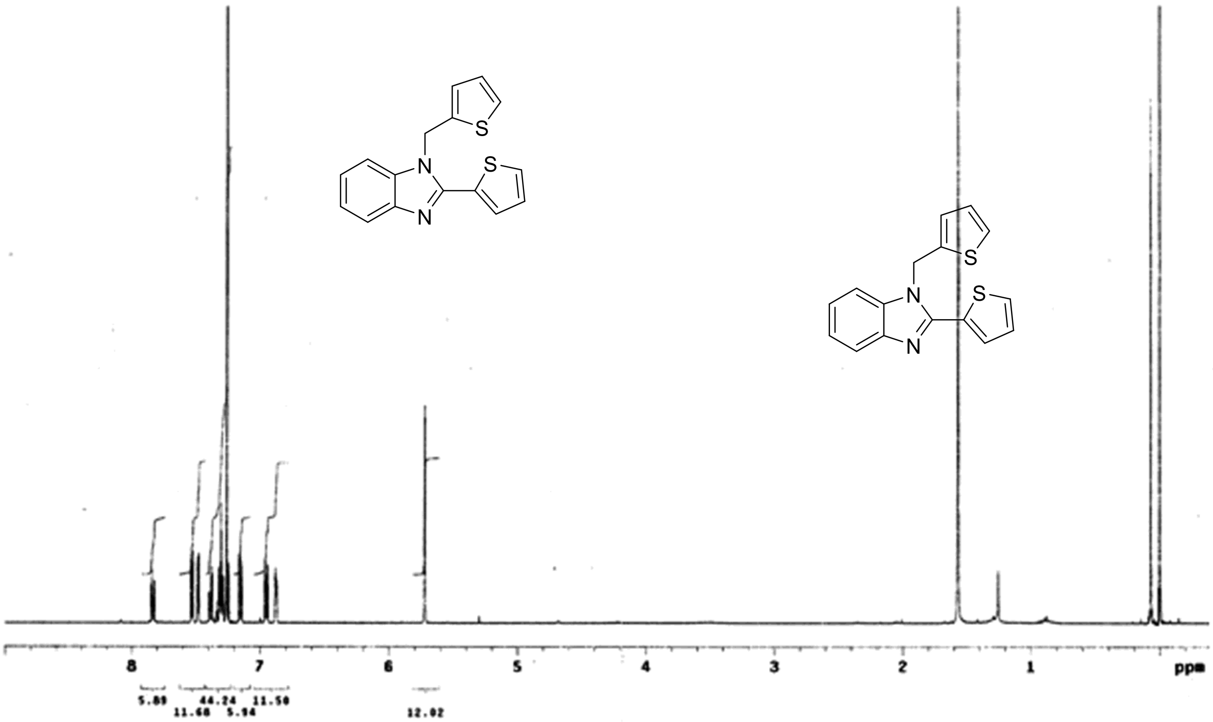

Fig. S18. ^{1}HNMR spectrum of **3q**.

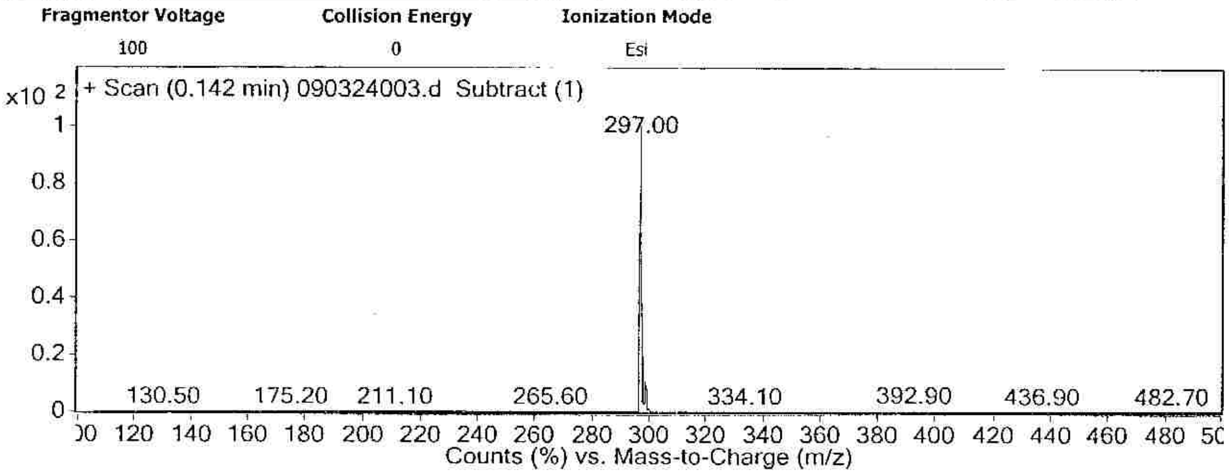

Fig. S19. Mass spectrum of **3q**.

Ethyl 6-methyl-2-oxo-4-phenyl-1,2,3,4-tetrahydropyrimidine-5-carboxylate (7a):

Off white solid, m.p. 207-209°C; ^{1}H NMR (400MHz, d_6-DMSO, δ/ppm): 9.19 (s, 1H, -NH), 7.75 (s, 1H, -NH), 7.34-7.31 (m, 2H, arom H), 7.26-7.23 (m, 3H, arom H), 5.15 (d, J = 3.2 Hz, 1H, -CH), 3.95 (q, J = 7.2 Hz, 2H, $-CH_2$), 2.25 (s, 3H, $-CH_3$), 1.09 (t, J = 7.2 Hz, 3H, $-CH_3$); ^{13}C NMR (100 MHz, d_6-DMSO): δ 165.83 (1C), 152.63 (1C), 148.81 (1C), 145.27 (1C), 128.87 (2C), 127.77 (1C), 126.70 (2C), 99.75 (1C), 59.70 (1C), 54.40 (1C), 18.22 (1C), 14.52 (1C); HRMS: Anal. Calcd. For $C_{14}H_{17}O_3N_2$: 261.1234; Found: 261.1225.

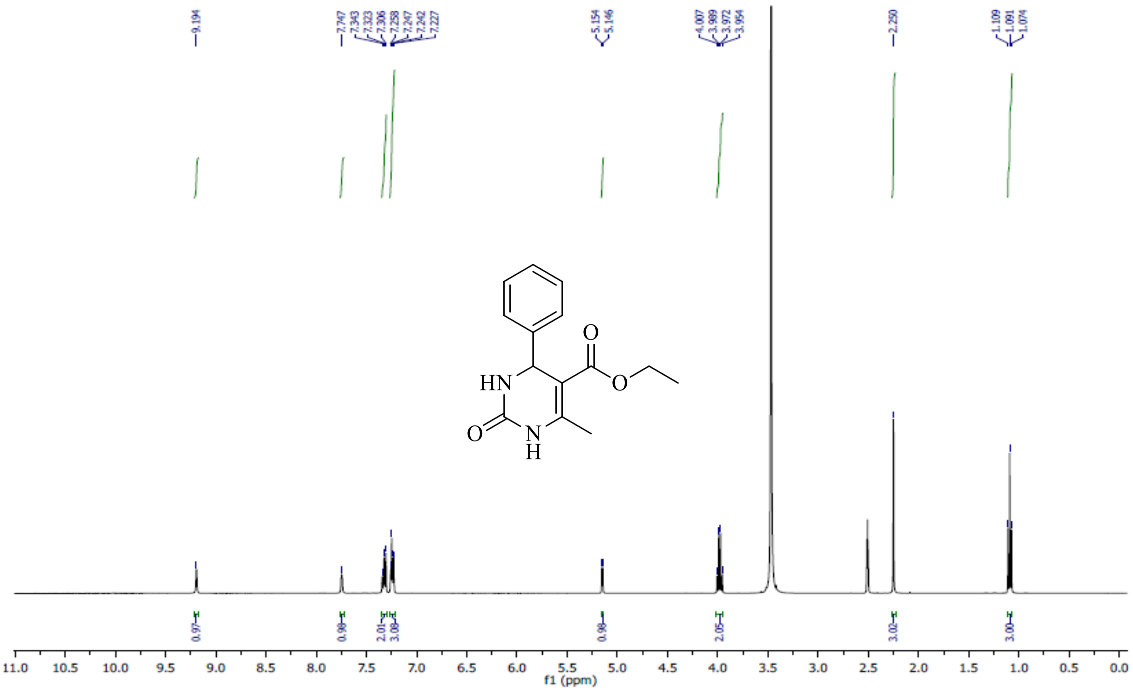

Fig. S20. ^{1}H NMR spectrum of **7a**.

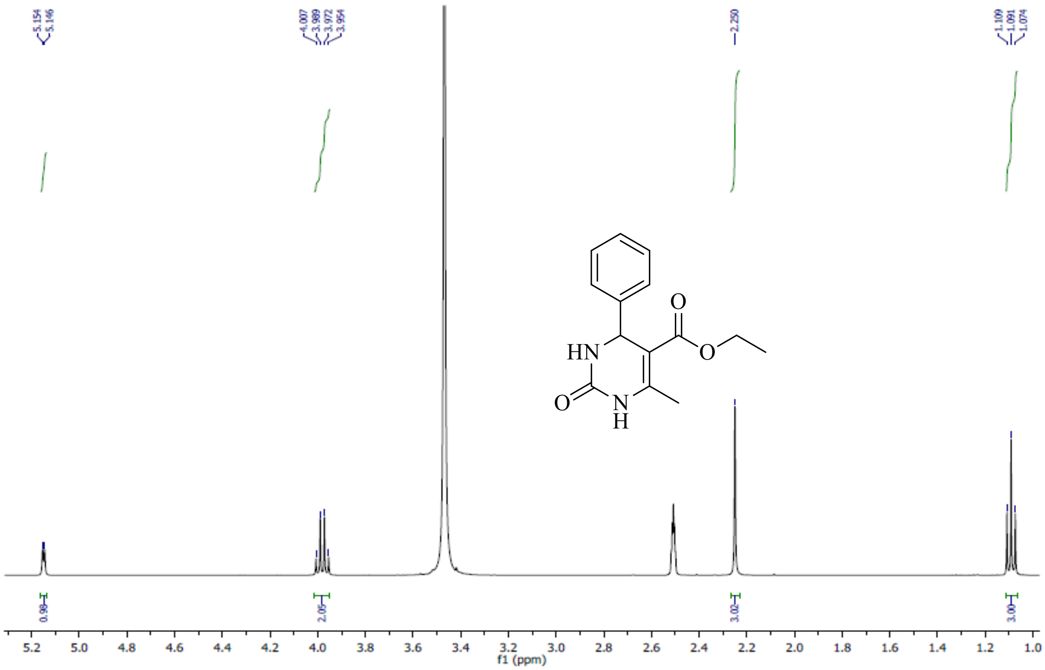

Fig. S21. Expanded ^{1}H NMR spectrum of **7a**.

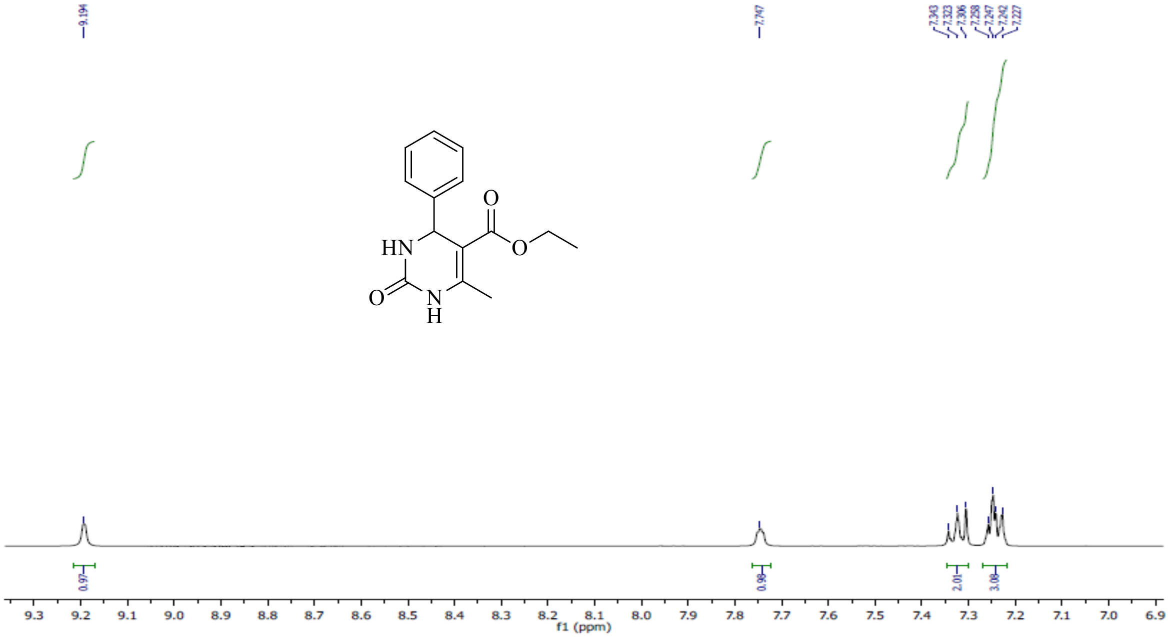

Fig. S22. Expanded ^{1}H NMR spectrum of **7a**.

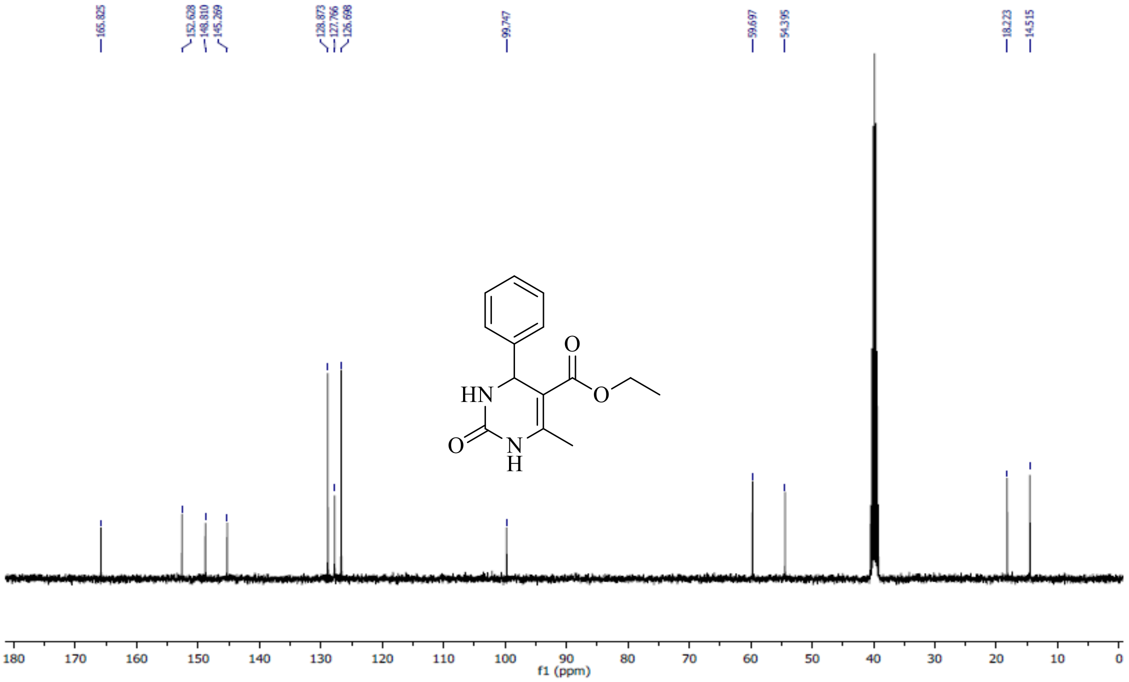

Fig. S23. ^{13}C NMR spectrum of **7a**.

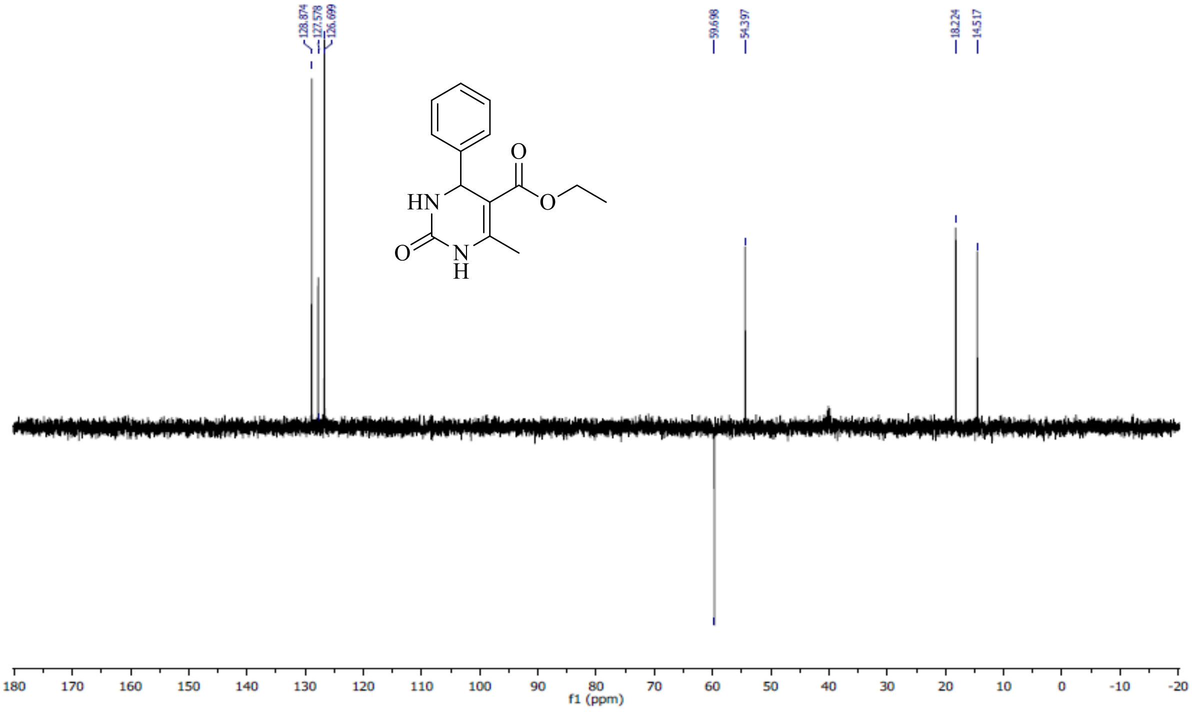

Fig. S24. DEPT-135 spectrum of **7a**.

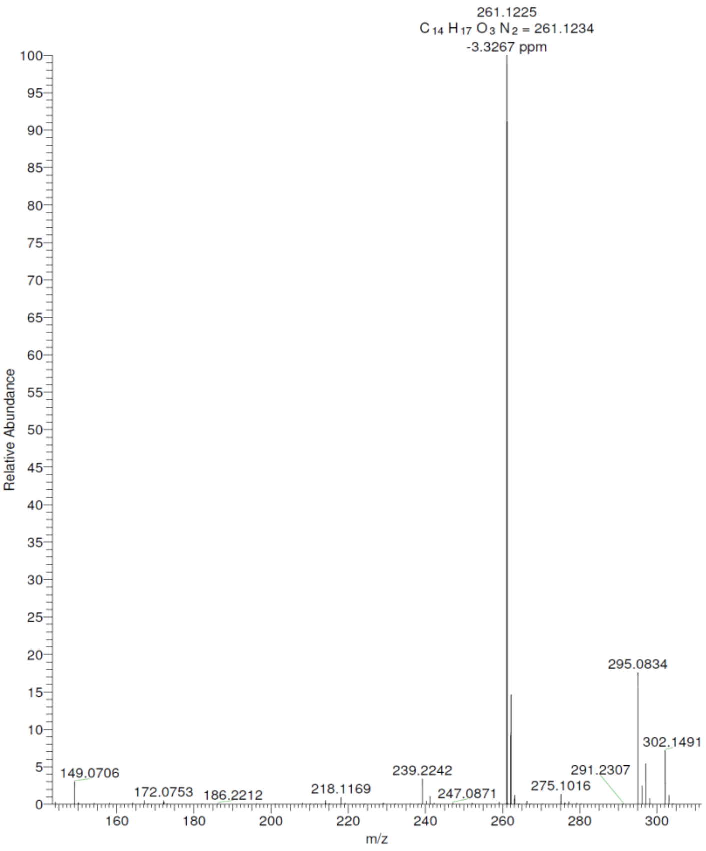

Fig. S25. HRMS spectrum of **7a**.

2-Amino-4-(3,4,5-trimethoxyphenyl)-6-(pyridine-2-ylthio)pyridine-3,5-dicarbonitrile (11c): White solid, m.p. 267-269°C; ^{1}H NMR (500 MHz, DMSO-d_6, δ/ppm): 8.58 (ddd, J = 4.5 Hz, J = 1.5 Hz, J = 1.0 Hz, 1H, arom H), 7.96 (brs, 2H, -NH_2), 7.88 (td, J = 8.0 Hz, J = 2.0 Hz, 1H, arom H), 7.80 (d, J = 8.0 Hz, 1H, arom H), 7.45-7.42 (m, 1H, arom H), 6.95 (s, 2H, arom H), 3.83 (s, 6H, -2OCH_3), 3.76 (s, 3H, -OCH_3).

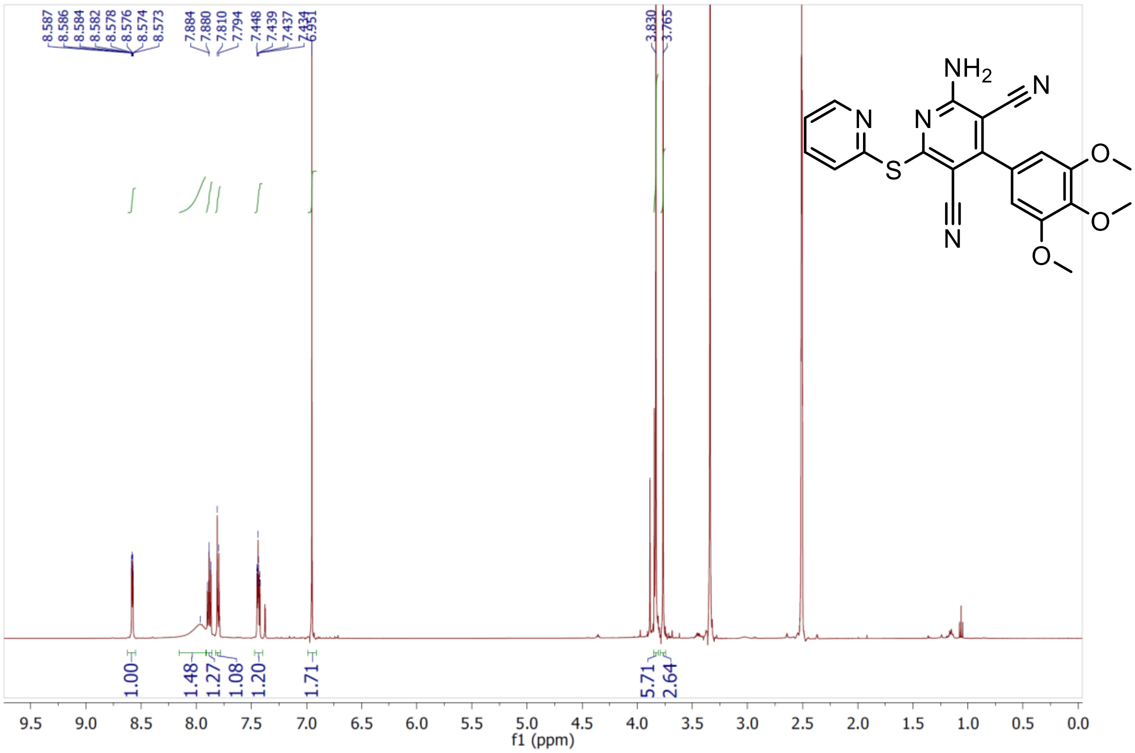

Fig. S26. ^{1}H NMR spectrum of **11c**.

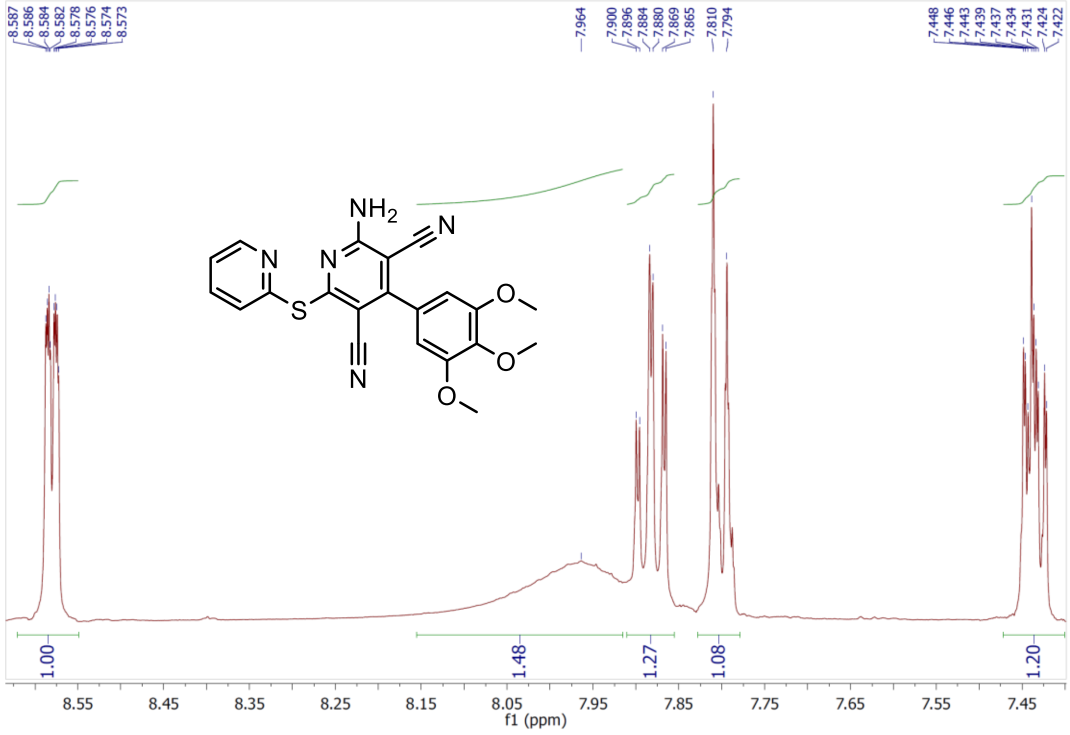

Fig. S27. Expanded ^{1}H NMR spectrum of **11c**.

2-Amino-4-(3-hydroxyphenyl)-6-(pyridine-2-ylthio)pyridine-3,5-dicarbonitrile (11d): White solid; m.p. 223-224°C; ^{1}H NMR (500 MHz, DMSO-d_6, δ/ppm): 9.87 (s, 1H, -OH), 8.54 (s, 1H, arom H), 7.92 (br, 2H, -NH_2), 7.85-7.76 (m,2H, arom H), 7.40-7.32 (m, 2H, arom H), 6.94-6.87 (m, 3H, arom H).

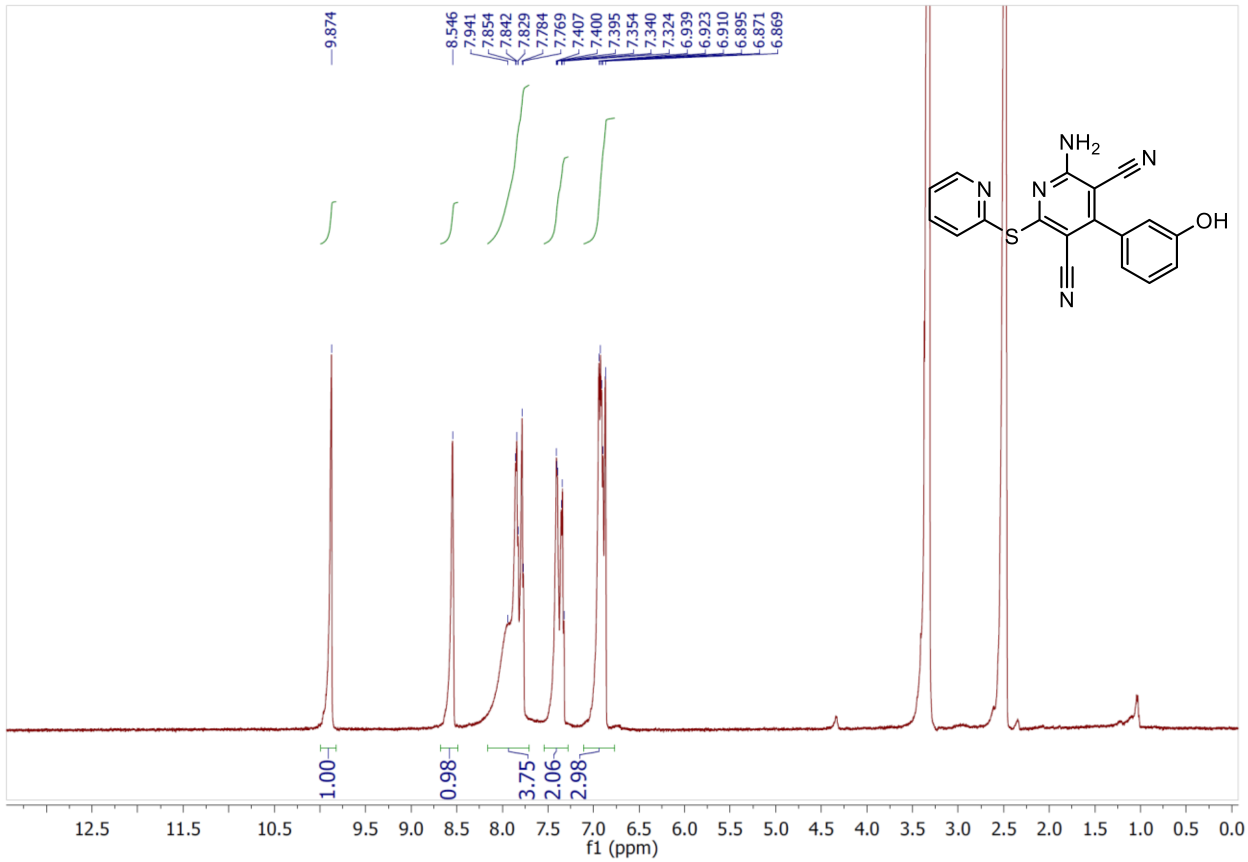

Fig. S28. ^{1}H NMR spectrum of **11d**.

2-Amino-4-(4-nitrophenyl)-6-(pyridine-2-ylthio)pyridine-3,5-dicarbonitrile (11e): White solid; Yield= 85%; m.p. 241-243°C; ^{1}H NMR (500 MHz, DMSO-d_6, δ/ppm): 8.58 (d, J = 6.5 Hz, 1H, arom H), 8.44 (d, J = 8.5 Hz, 2H, arom H), 8.12 (brs, 2H, -NH_2), 7.89 (d, J = 8.5 Hz, 2H, arom H), 7.87 (d, J = 1.5 Hz, 1H, arom H), 7.81 (d, J = 8.0 Hz, 1H, arom H), 7.44 (dd, J = 7.5 Hz, J = 5.0 Hz, 1H, arom H).

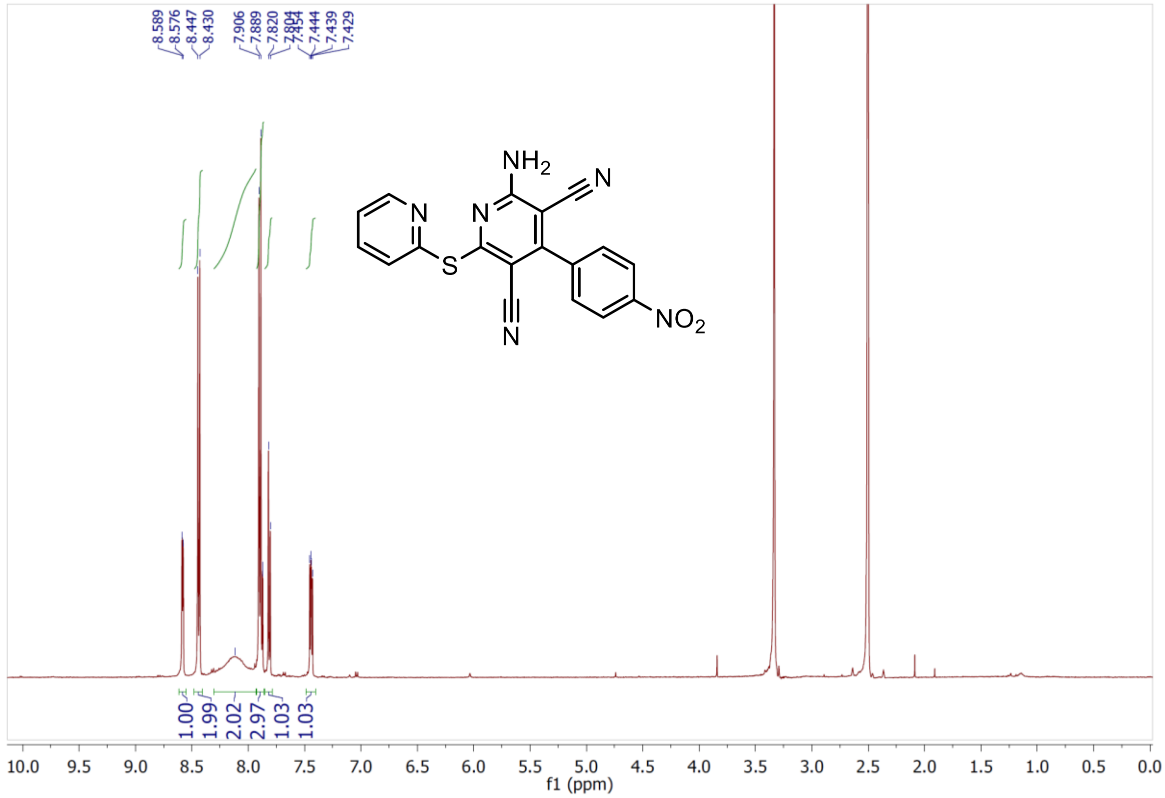

Fig. S29. ^{1}H NMR spectrum of **11e**.

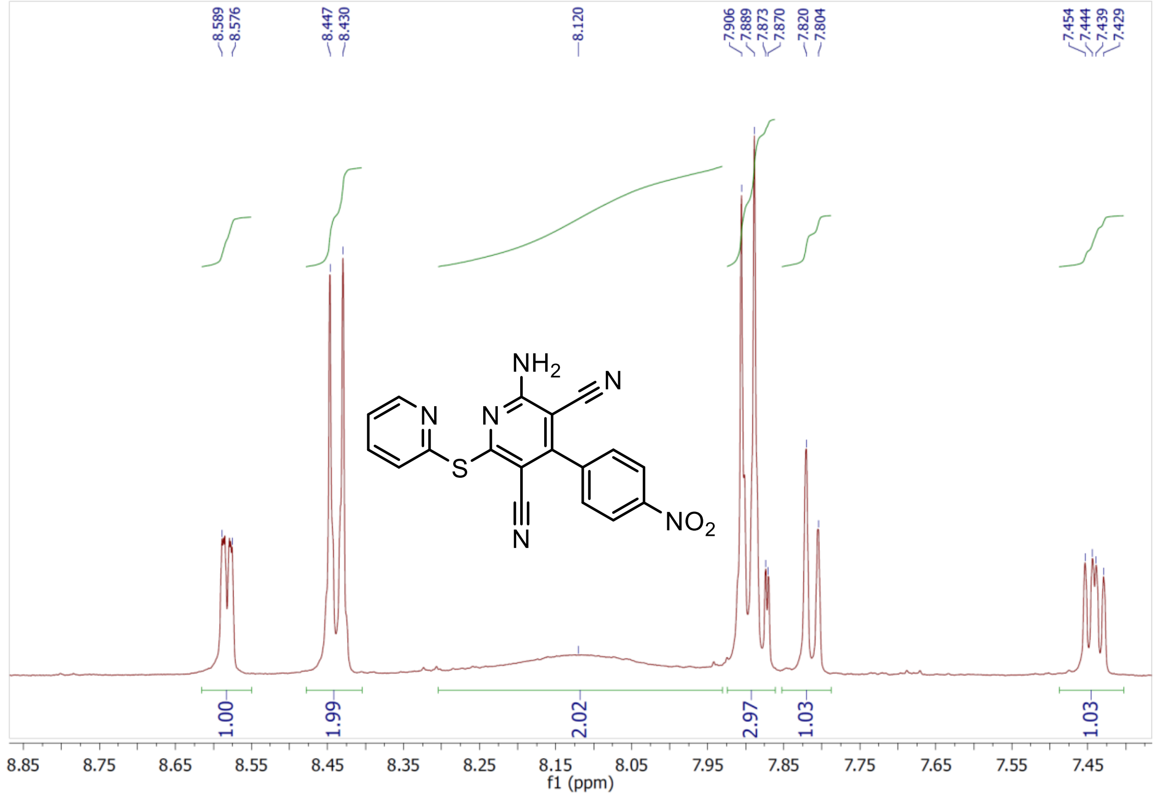

Fig. S30. Expanded ^{1}H NMR spectrum of **11e**.

2-Amino-4-(3, 4-difluorophenyl)-6-(pyridine-2-ylthio)pyridine-3,5-dicarbonitrile (11h):

White solid; m.p. 252-253°C; ^{1}H NMR (500 MHz, DMSO-d_6, δ/ppm): 8.58 (dd, *J*= 5.0 Hz, *J*= 1.0 Hz, 1H, arom H), 8.06 (br, 2H, -NH_2), 7.88 (td, *J* = 8.0 Hz, *J*=2.0 Hz, 1H, arom H), 7.83 (td, *J* = 7.5 Hz, *J*=2.0 Hz, 1H, arom H), 7.80 (d, *J*= 8.0 Hz, 1H, arom H), 7.70 (dt, *J* = 8.5 Hz, *J*=2.0 Hz,1H, arom H), 7.83 (ddd, *J* = 7.5 Hz, *J* = 5.0 Hz, *J*=1.0 Hz, 1H, arom H).

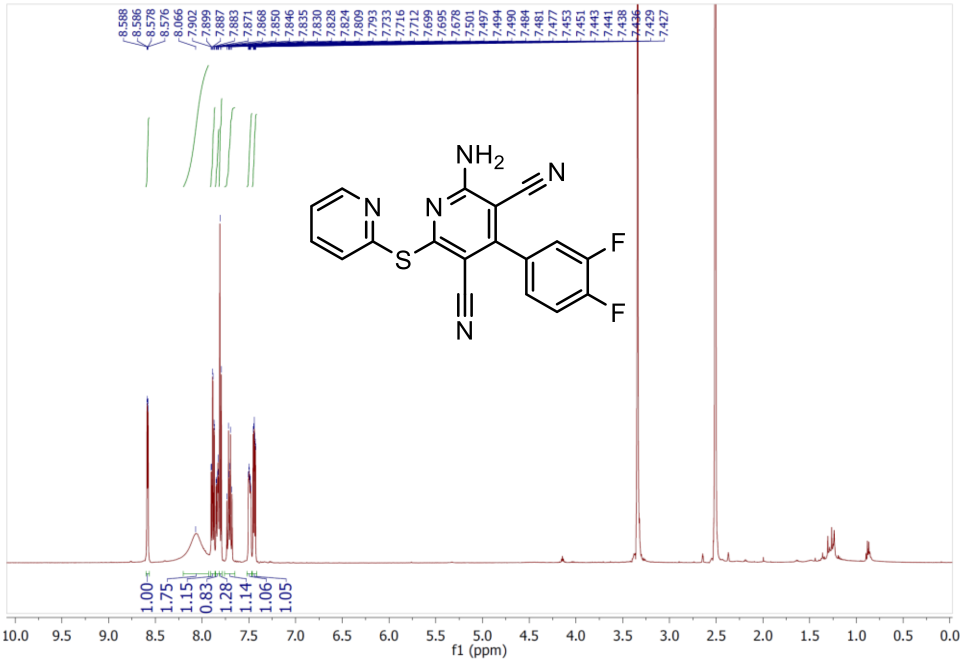

Fig. S31. ^{1}H NMR spectrum of **11h**.

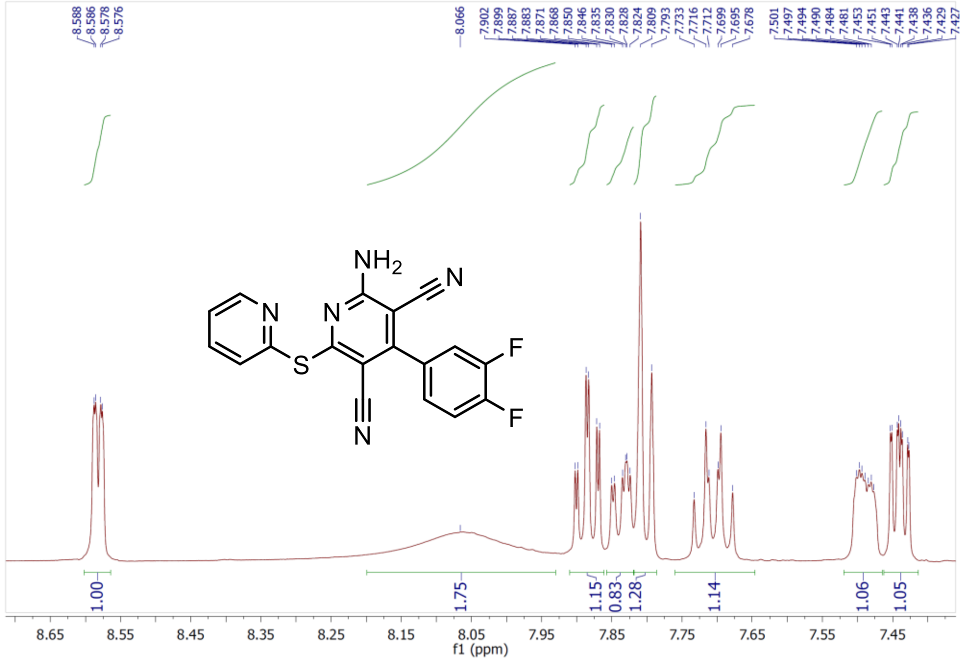

Fig. S32. Expanded ^{1}H NMR spectrum of **11h**.

6-Amino-4-(pyridin-2-ylthio)-[2,4'-bipyridine]-3,5-dicarbonitrile (**11i**): White solid; m.p. 230-231°C; ^{1}H NMR (500 MHz, DMSO-d_6, δ/ppm): 8.82 (dd, *J* = 4.5 Hz, *J* = 2.0 Hz, 2H, arom H), 8.58 (ddd, *J* = 5.0 Hz, *J* = 2.0 Hz, *J* = 1.0 Hz, 1H, arom H), 8.1 (brs, 2H, -NH_2), 7.88 (td, *J* = 8.0 Hz, *J* = 2.0 Hz, 1H, arom H), 7.81 (d, *J* = 8.5 Hz, 1H, arom H), 7.61 (dd, *J* = 4.5 Hz, *J* = 2.0 Hz, 2H, arom H), 7.44 (ddd, *J* = 7.0 Hz, *J* = 4.5 Hz, *J* = 1.0 Hz, 1H, arom H).

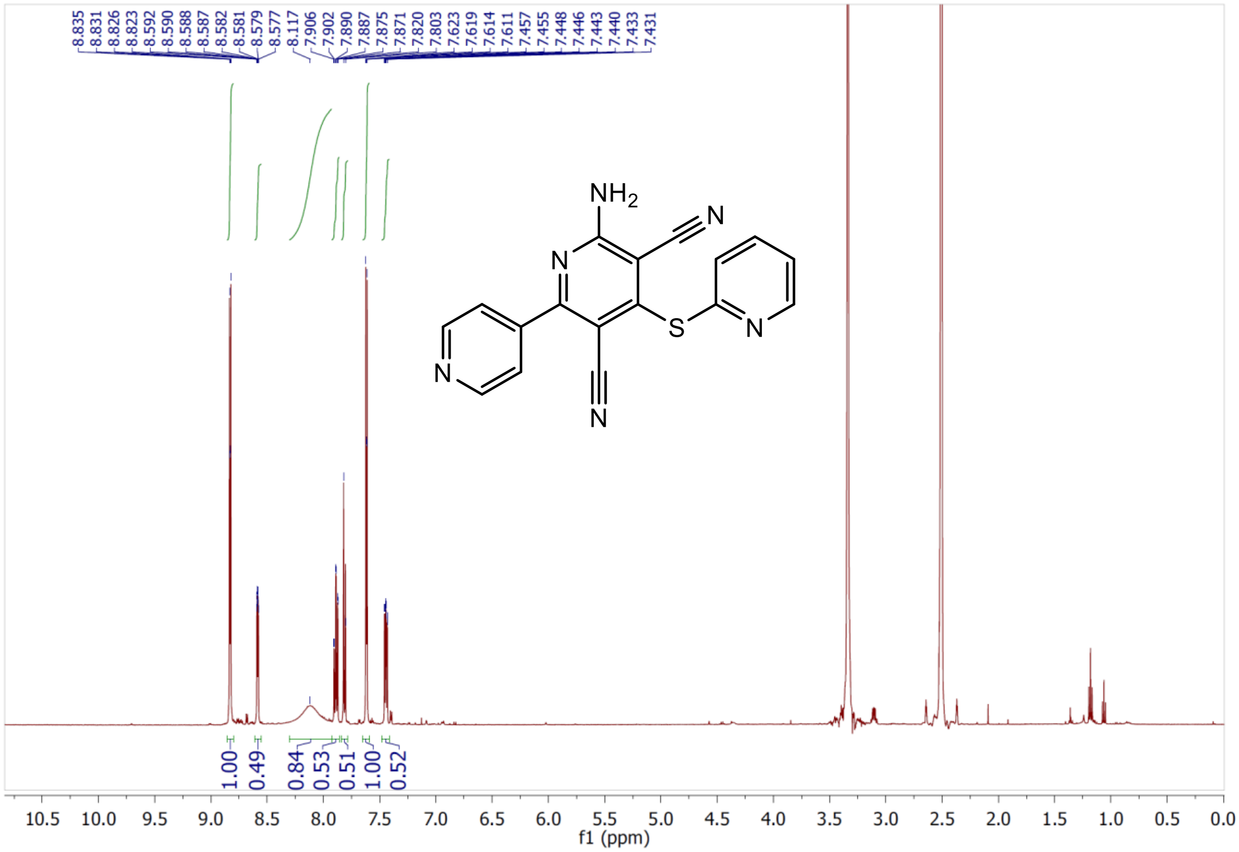

Fig. S33. ^{1}H NMR spectrum of **11i**.

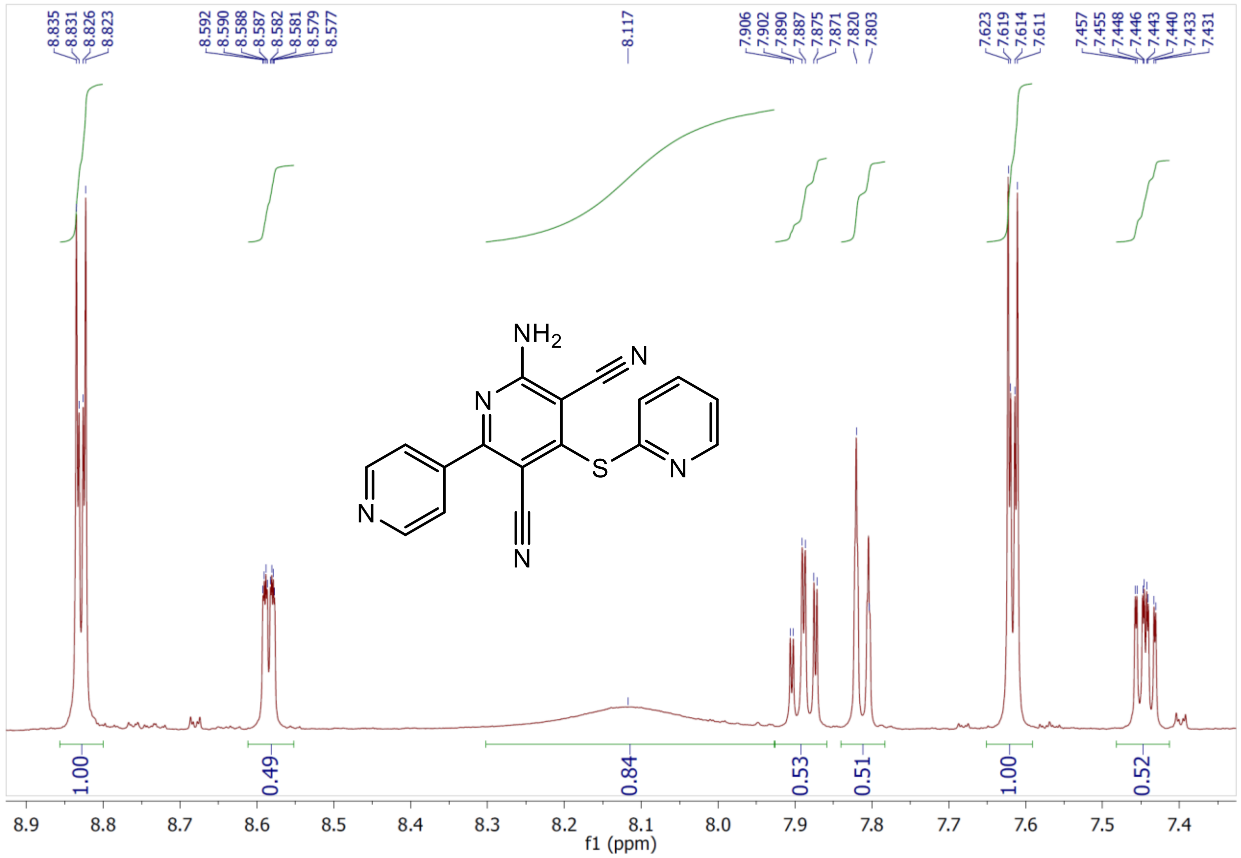

Fig. S34. Expanded ^{1}H NMR spectrum of **11i**.

Detailed calculations of the green chemistry metrics for the synthesis of the compounds 3a, 7a, and 11a

Reaction-S1: Green chemistry metrics for the synthesis of **3a**.

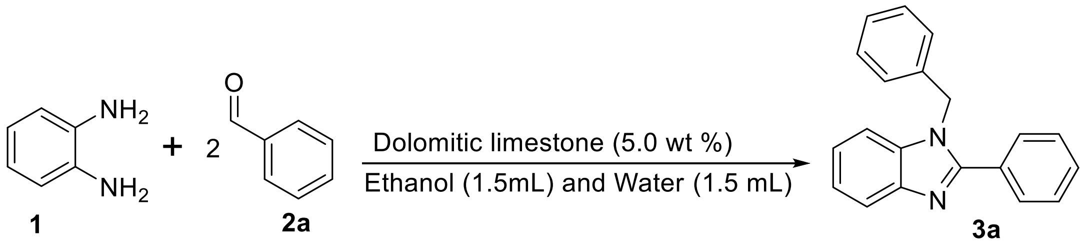

	1	2a	3a
Mass	0.108 g	0.212 g	0.279 g
Moles	0.001 mole	0.002 mole	0.00098 mole
GMW	108	106 x 2 = 212	284

Catalyst mass: 0.017 g; **Mass of recovered materials**: 0.017 g;

Reaction Solvent Mass: 2.68g [Ethanol (1.5mL) and Water (1.5 mL)]

Mass of Work-up Material: 4.5 g [Ethylacetate (5 mL)]

Mass of Purification Material: 4.18 g [Ethanol (1.5 mL) and Water (3 mL)]

Total input mass (^{m}inputs) = ^{m}1 + ^{m}2a + ^{m}solvent (S) + ^{m}catalyst (C) + ^{m}workup materials (WPM) + ^{m}purification materials (PM) = 0.108 + 0.212 + 2.68 + 0.017 + 4.5 + 4.18 = 11.697

Evaluation of the green chemistry metrics

Atom Economy(AE, %) = 100 (GMW of product / Sum of GMWs of reactants)

= 100 (284/108+212) = 89%

E-factor (E) =Total input mass (^{m}inputs) - Mass of target product (^{m}3a) - Mass of recovered materials/Mass of target product (^{m}3a)

= 11.697- 0.279 - 0.017/0.279 = 40.864

Process Mass Intensity (PMI) = (^{m}inputs - Mass of recovered materials)/^{m}3a

= 11.697 - 0.017/0.279 = 41.864

(Or)

= 1 + E = 1 + 40.864= 41.864

Curzon's Reaction Mass Efficiency (Curzon's RME, %)

= 100 (mass of **3a**/mass of **1** + mass of **2a)**

= 100 (0.279 /0.108 + 0.212) = 87%

(Or)

=100 (Yield of 3a x Atom Economy x 1/Stoichiometric Factor)

= 100(0.98 x 0.89 x 1) = 87% (∵ Stoichiometric Factor (SF) = 1)

Generalized Reaction Mass Efficiency (gRME, %)

= 100 [^{m}3a/(^{m}inputs - Mass of recovered materials)]

=100[0.279/11.697 - 0.017] =2.4%

(Or)

= 100[1/(1+E)] = 100[1/1 + 40.864] = 2.4%

Reaction-S2: Green chemistry metrics for the synthesis of **7a**.

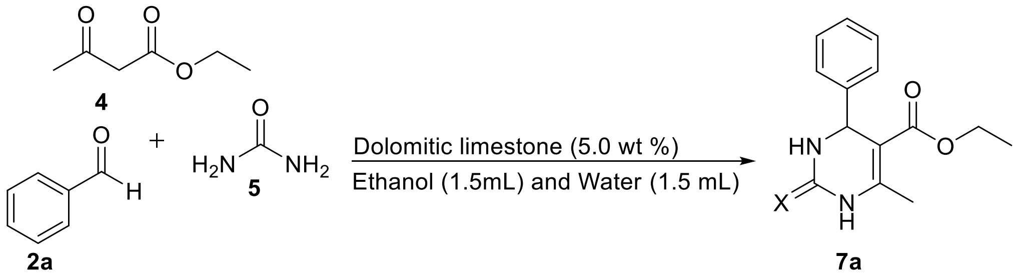

	**2a**	**4**	**5**	**7a**
**Mass**	0.106 g	0.13 g	0.06 g	0.252 g
**Moles**	0.001 mole	0.001 mole	0.001 mole	0.00097 mole
**GMW**	106	130	60	260

Catalyst mass: 0.0155 g; **Mass of recovered materials**: 0.0155 g;

Reaction Solvent Mass: 2.68g [Ethanol (1.5mL) and Water (1.5 mL)]

Mass of Work-up Material: 4.5 g [Ethylacetate (5 mL)]

Mass of Purification Material: 4.18 g [Ethanol (1.5 mL) and Water (3 mL)]

Total input mass (^{m}inputs) = ^{m}**2a** + ^{m}**4** + ^{m}**5** + ^{m}solvent (S) + ^{m}catalyst (C) + ^{m}workup materials (WPM) + ^{m}purification materials (PM) = 0.106 + 0.13 + 0.06 + 2.68 + 0.0155 + 4.5 + 4.18 = 11.6715

Evaluation of the green chemistry metrics

Atom Economy (AE, %) = 100 (GMW of product / Sum of GMWs of reactants)

= 100 (260/106+130+60) = 88%

E-factor (E) = Total input mass (^{m}inputs) - Mass of target product (^{m}**7a**) - Mass of recovered materials/Mass of target product (^{m}**7a**)

= 11. 6715 - 0.252 - 0.0155/0.252 = 45.254

Process Mass Intensity (PMI) = (^{m}inputs - Mass of recovered materials)/^{m}**7a**

= 11. 6715 - 0.0155/0.252 = 46.254

(Or)

= 1 + E = 1 + 45.254 = 46.254

Curzon's Reaction Mass Efficiency (Curzon's RME, %) = 100 (mass of **7a**/mass of **2a** + mass of **4 +** mass of **5)**

= 100 (0.252 /0.106 + 0.13 + 0.06) = 85%

(or)

=100 (Yield of **7a** x Atom Economy x 1/Stoichiometric Factor)

= 100 (0.97 x 0.88 x 1) = 85% (∵ Stoichiometric Factor (SF) = 1)

Generalized Reaction Mass Efficiency (gRME, %) = 100 [^{m}**7a**/(^{m}inputs - Mass of recovered materials)]

=100[0.252/11.6715- 0.0155] = 2.2%

(Or)

= 100[1/(1+E)] = 100[1/1 + 45.254] = 2.2%

Reaction-S3: Green chemistry metrics for the synthesis of **11a**.

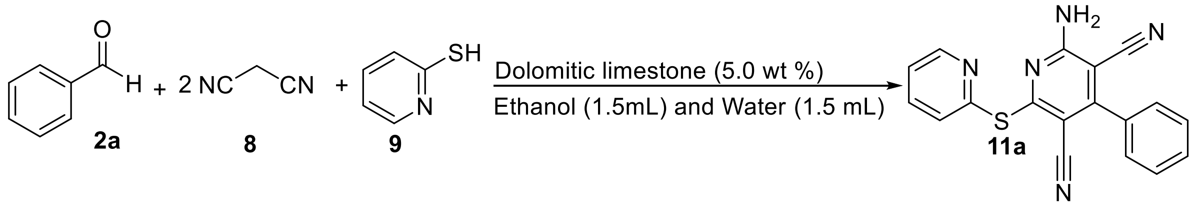

	**2a**	**8**	**9**	**11a**
**Mass**	0.106 g	0.132 g	0.111 g	0.316 g
**Moles**	0.001 mole	0.002 mole	0.001 mole	0.00096 mole
**GMW**	106	66 x 2 = 132	111	329

Catalyst mass: 0.0185 g; **Mass of recovered materials**: 0.0185 g;

Reaction Solvent Mass: 2.68g [Ethanol (1.5mL) and Water (1.5 mL)]

Mass of Work-up Material: 4.5 g [Ethylacetate (5 mL)]

Mass of Purification Material: 4.18 g [Ethanol (1.5 mL) and Water (3 mL)]

Total input mass (^{m}inputs) = ^{m}**2a** + ^{m}**8** + ^{m}**9** + ^{m}solvent (S) + ^{m}catalyst (C) + ^{m}workup materials (WPM) + ^{m}purification materials (PM) = 0.106 + 0.132 + 0.111+ 2.68 + 0.0185 + 4.5 + 4.18 = 11.7275

Evaluation of the green chemistry metrics

Atom economy (AE, %) = 100 (GMW of product / Sum of GMWs of reactants)

= 100 (329/106+132+111) = 94%

E-factor (E) = Total input mass (^{m}inputs) - Mass of target product (^{m}**11a**) - Mass of recovered materials/Mass of target product (^{m}**11a**)

= 11.7275 - 0.316 - 0.0185/0.316 = 36.054

Process mass intensity (PMI)

= (^{m}inputs - Mass of recovered materials)/^{m}**11a**

= 11.7275 - 0.0185/0.316 = 37.054

(Or)

= 1 + E = 1 + 36.054 = 37.054

Curzon's reaction mass efficiency (Curzon's RME, %)

= 100 (mass of **11a**/mass of **2a** + mass of **8 +** mass of **9)**

= 100 (0.316 /0.106 + 0.132 + 0.111) = 90%

(or)

=100 (Yield of **11a** x Atom Economy x 1/Stoichiometric Factor)

= 100 (0.96 x 0.94 x 1) = 90% (∵ Stoichiometric Factor (SF) = 1)

Generalized reaction mass efficiency (gRME, %)

=100 [^{m}**11a**/(^{m}inputs - Mass of recovered materials)]

= 100[0.316/11.7275 - 0.0185] = 2.7%

(Or)

= 100[1/(1+E)] = 100[1/1 + 36.054] = 2.7%
